# Raman-guided sample subset selection for cost-efficient offline calibration in bioprocesses

**DOI:** 10.1007/s00449-026-03402-x

**Published:** 2026-08-12

**Authors:** Terrance Wilms, Fabian Schwenke, Rudibert King, Steffi Knorn

**Affiliations:** Chair of Control, Technische Universität Berlin, Secr. ER 2-1, Hardenbergstraße 36a, 10623 Berlin, Germany

**Keywords:** Sample subset selection, PAT, Nonlinear parameter identification, Raman spectroscopy, Bioprocess modeling

## Abstract

In bioprocess engineering, model-based methods play a vital role in understanding complex dynamics of novel species or strains. However, the model development is often hampered by prohibitive costs associated with redundant reference analyses and poorly informed sampling schedules due to insufficient prior knowledge about the process dynamics. We propose a model-free PAT strategy, termed Raman-guided sample subset selection (RGSS) to prioritise informative offline reference assays from inline Raman spectra before nonlinear model calibration. RGSS is demonstrated with *Saccharomyces cerevisiae* fed-batch fermentations and evaluated by auto- and cross-validation (CV), as well as with practical identifiability analysis of an unstructured mechanistic model. The approach is implemented using constrained vector quantization (CVQ) with full-spectrum and analyte-specific wavenumber-selection and benchmarked against uniform-in-time sampling, random subsampling, and Kennard–Stone (KS) selection. The best RGSS scenarios, using only five selected reference assays per analyte, retained offline CV accuracy (NRMSE = 3.49) close to the full-data reference (NRMSE = 3.29) using all 22 samples. KS selection was also competitive for ten selected samples (NRMSE = 3.79), whereas uniform-in-time sampling resulted in higher CV errors for five and ten samples (8.47 and 65.57). Random subsampling occasionally produced competitive subsets, but showed broad variability over 20 random runs (NRMSE median [IQR]: 6.14 [4.84–47.76] and 15.48 [5.07–44.34]) for five and ten randomly selected samples. These results support RGSS as a cost-efficient, spectrally informed sample subset selection framework for prioritising offline assays in model-based bioprocess development.

**Supplementary Information:**

The online version contains supplementary material available at https://doi.org/10.1007/s00449-026-03402-x.

## Introduction

As process systems engineering continues to evolve, it is essential to utilize its potential to address current challenges to shape the future of chemical and bioprocess engineering and technological processes [1]. In this context, microbial fermentation is expected to play a pivotal role, particularly with the rising trend towards plant-based foods and sustainable chemicals. New startups are utilizing cutting-edge technologies to drive innovation and expand the market [2]. For instance, EuroAPI, a company initiated by Sanofi, focuses on fermentation-based manufacturing to reduce Europe’s reliance on external supply [3]. Model-based methods are essential for understanding, scaling, and controlling modern bioprocesses [4, 5], especially when new strains or process conditions are explored [6–9]. During early research and development (R&D), extensive data generation is crucial for building reliable mechanistic models [10]. However, taking and analysing dense offline sampling schemes is often economically and logistically infeasible [11]. This challenge is amplified by the scale of biopharmaceutical development, where R&D efforts have been reported to exceed $50 million and 200 person-years over 5–10 years [12]. Consequently, model development, particularly parameter identification (PI), may remain practically unreliable due to sparse high-quality calibration data.

At the same time, modern automated sampling and at-line analyser platforms can shift the bottleneck from taking samples to deciding which of them should undergo costly reference analytics [13–15]. In such settings, systematic prioritisation of informative samples becomes crucial for cost-efficient model development. In parallel, optimal experimental design (OED) or model-based design of experiments (mbDoE) are established strategies to maximize information content for PI, often using sensitivity- and Fisher Information Matrix (FIM)-based criteria [16, 17], bootstrap analysis [18] or unscented transformation [19]. For dynamic bioprocess models, these methods typically optimize sampling times and input profiles to improve parameter precision [16, 17, 20]. However, all of these OED methods require a sufficiently parameterized model and may be inherently affected by plant–model mismatch (PMM), i.e., unavoidable discrepancies between the true process dynamics and the assumed model structure [21]. Because sampling times and inputs are optimized under the assumed model dynamics, structural inaccuracies can lead to suboptimal designs. Hence, a model-free empirical method is highly beneficial as a sample subset selection strategy in the pre-model stages, where both parameters and model structure are largely unknown.

Process analytical technology (PAT) and spectroscopy offer attractive routes to reduce experimental effort by providing rich online information. Among spectroscopy, Raman spectroscopy provides distinct molecular fingerprints and is less affected by water absorption than near- and mid-infrared (NIR/MIR) spectroscopy, which is advantageous for aqueous fermentations [22–25]. These properties make Raman particularly suitable for ranking candidate offline sampling points in time based on spectral novelty and biochemical transitions. However, Raman spectra acquired in bioprocesses are affected not only by molecular concentration changes, but also by process- and measurement-related effects such as fluorescence background, turbidity, scattering, bubbles, probe fouling, and instrumental drift. Spectral preprocessing is therefore an important step in Raman-based PAT and chemometric workflows, because baseline correction, normalization, smoothing, spike correction, and derivative-based transformations can strongly influence the extracted spectral information and the subsequent multivariate analysis [26–28]. In this study, we deliberately use a minimal preprocessing strategy based on airPLS baseline correction only. This choice avoids introducing an additional preprocessing-optimization layer or systematic preprocessing-configuration screening and keeps the comparison between spectral subset-selection strategies interpretable.

Recent discussions have also highlighted that, despite two decades of PAT initiatives, analytical and modelling workflows still face significant complexity and resource constraints [11]. We therefore employ Raman spectra as the information source to rank and select candidate offline sampling points in time. Spectroscopy has traditionally been applied in biotechnology and pharmaceutical manufacturing as a PAT for predicting immeasurable process variables [29–31]. The capabilities of UV/Vis, IR, fluorescence, and Raman spectroscopy can be maximized by integrating these spectroscopic techniques with multivariate data analysis or by applying physically based spectral models, thereby extracting valuable process information from large datasets [32]. The initial utilization of Raman spectroscopy for online monitoring in yeast fermentation was pioneered by Shaw et al. [33]. Subsequently, Raman spectroscopy found broader application for the indirect and on-line measurement of process variables in this context [34]. Yang et al. [35] recently developed an in-situ monitoring calibration model based on Raman spectroscopy, employing principal component analysis (PCA) for dimensionality reduction and spectral exploration, combined with partial least squares (PLS) regression to predict concentrations in a mixed solution within a complex matrix. In addition, spectroscopic data contain valuable insights into molecular changes, combinations, variations, and interactions, revealing the quality of samples. By extracting this information and ranking the samples from high to low information-rich spectral data, we can select the most suitable samples for reference analysis. A method utilizing Constrained Vector Quantization (CVQ) [36, 37] for spectroscopic data has demonstrated the ability to select a smaller data set with almost the same information content as the original set of raw data [38]. More broadly, sample subset selection is an established problem in chemometrics and multivariate modelling, where representative subsets are chosen from large unlabelled datasets to reduce the cost and time of reference analyses while retaining information content of the original data [39]. Such methods can be broadly categorized into distance-based, clustering-based, experimental-design-inspired, and kernel-based approaches. Among these, Kennard–Stone (KS) algorithm is one of the most widely used distance-based methods and sequentially selects samples that maximize the spread in the spectral space [40]. Random sampling is commonly used as a simple baseline, but it does not guarantee representative coverage of the original data, particularly for small subset sizes [39, 41, 42]. CVQ represents a less established but promising kernel-based alternative, where representative subsets are selected from the measured data by minimizing a mean-discrepancy objective [36–38].

In this contribution, we extend the concept of sample subset selection to Raman-guided offline assay prioritization in bioprocess model development. In contrast to conventional calibration-set partitioning, the goal is not only to split an existing labelled dataset, but to decide which stored candidate samples should undergo costly offline reference analytics before nonlinear PI of a mechanistic model. We therefore propose Raman-guided sample subset selection (RGSS) and evaluate both full-spectrum and analyte-specific wavenumber-selected CVQ and KS variants. To place these spectral strategies into the broader context of chemometric subset selection, we additionally benchmark them against random subsampling and uniform-in-time sampling.

The performance and uncertainty of mechanistic bioprocess models critically depend on the quantity and quality of calibration data [43]. PI often involves nonlinear optimizations, for which numerous methodologies are documented in the literature. To this end, Villaverde et al. [44] conducted a comprehensive analysis, highlighting the superior performance of a hybrid approach integrating multi-start of interior-point optimization and a global metaheuristic, particularly when applied to extensive kinetic models. In this study, we use a similar metaheuristic presented in [45] that combines Latin hypercube sampling (LHS) [46, 47] (for multiple initialization) with a genetic algorithm (GA) and gradient-based local refinement. Following PI, a sensitivity-based parameter subset selection (SbS) [48] is applied to verify practical identifiability, as demonstrated by [21, 49], who address identifiability challenges in ill-posed problems using SbS.

*Saccharomyces cerevisiae* (*S. cerevisiae*) is used as a case study, because it is a well-established platform organism in industrial biotechnology and metabolic engineering [50, 51]. RGSS is applied to two fed-batch fermentations. F1 is used for PI and auto-validation (AV), whereas F2 serves as an independent cross-validation (CV) experiment. CVQ and KS are evaluated using either full-spectrum feature matrices (FS-CVQ, FS-KS) or analyte-specific wavenumber-selected feature matrices (WS-CVQ, WS-KS). For FS-based strategies, one common subset is selected for all offline analytes. For WS-based strategies, the nominal subset size denotes the number of selected reference assays per analyte. Random subsampling (RS) and uniform-in-time (UI) sampling are included as non-spectral baselines.

To the best of our knowledge, RGSS is one of the first approaches in bioprocess engineering that uses spectroscopic data, instead of a pre-existing mechanistic model, to prioritise informative offline samples for dynamic model calibration. The main contributions of this study are:

- A model-free Raman-guided sample subset selection framework for prioritizing informative offline reference assays before nonlinear PI.
- A comparison of full-spectrum and analyte-specific wavenumber-selected RGSS variants with CVQ and KS for biomass, glucose, ammonium and phosphate in *S. cerevisiae*.
- A benchmark against random and uniform-in-time subsampling to assess the value of spectrally informed RGSS.
- An evaluation of reduced calibration sets by AV, independent CV, NRMSE and sensitivity-based practical identifiability analysis.

Raman spectra are used exclusively as a high-throughput, information-rich basis for prioritizing a small number (subset) of offline samples for reference analytics. They are not used as additional measurement signals in a mechanistic state-estimation framework. Combining RGSS with Raman-based soft sensing or state-estimation is a promising extension, but is outside the scope of this work.

## Materials and methods

**Fig. 1:**
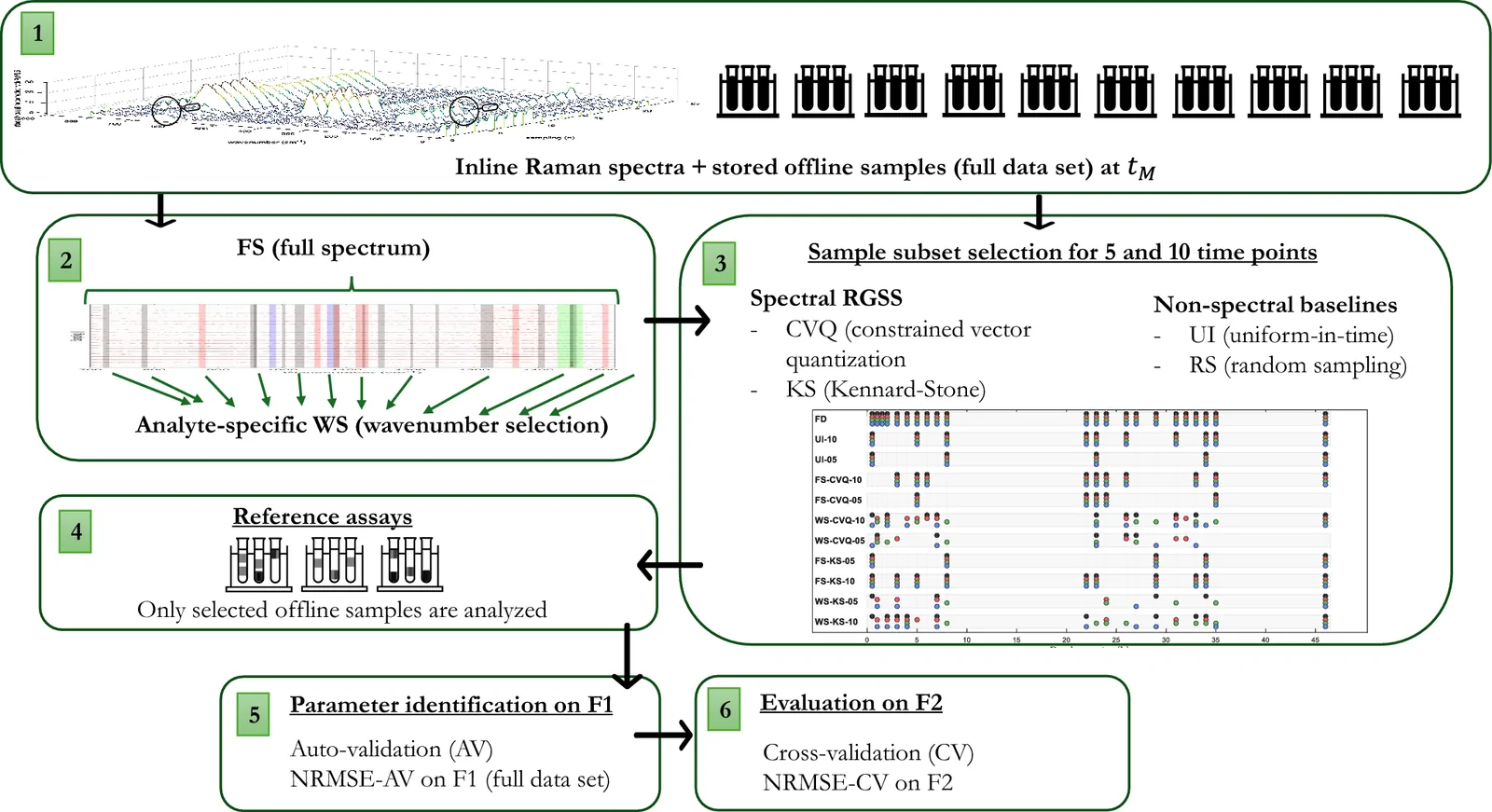
Workflow of the Raman-guided sample subset selection framework. Inline Raman spectra and stored offline samples form the full data set. Two spectral based strategies are considered: full-spectrum (FS) and analyte-specific wavenumber selection (WS). These spectral based sample subset-selection methods, i.e., constrained vector quantization (CVQ) and Kennard–Stone (KS), are compared with non-spectral baselines, i.e., uniform-in-time sampling (UI) and random subsampling (RS), for selecting 5 or 10 samples per analyte. Only the selected offline samples are analysed by reference assays and used for nonlinear parameter identification (PI) on F1. The resulting parameter estimates are evaluated by auto-validation (AV) on F1 (with full data set) and cross-validation (CV) on the independent F2 experiment. The workflow illustrates how inline Raman spectra can guide the selection of informative offline samples to reduce reference analytics while retaining model identification quality

Figure 1 summarizes the proposed RGSS workflow and the baseline comparison. First, inline Raman spectra are recorded throughout the fermentations and linked to candidate offline sampling time points $t_{_\mathrm{M}}$. Second, spectral feature matrices are defined either as full-spectrum (FS) representations or as analyte-specific wavenumber-selected (WS) representations. Third, sample subset selection is performed using CVQ or KS, while uniform-in-time and random subsampling are used as non-spectral baselines. Only the selected samples undergo offline reference analytics to generate a reduced calibration dataset. This dataset is used for nonlinear PI on F1 and evaluated by AV using the full-data (FD) set of F1. The resulting parameter set is then tested on the independent F2 experiment by CV using NRMSE.

### Experimental setup

The strain used for this study was *S. cerevisiae* (DSM 1334) obtained from Deutsche Sammlung von Mikroorganismen und Zellkulturen GmbH (DSMZ). The procedure followed for strain preparation, fermentation and analytic was in accordance with [52, 53].

The strain was stored at – 80 $^{\circ }$C and aliquots for fermenter cultivations were prepared at the Chair of Control, TU Berlin. To initiate the cultivations, cells were first grown in a shake flask at 30 $^\circ$C with a pH of 6, stirred at 120 rpm. The preculture medium recommended by DSMZ 186 consisted of yeast extract (3 g/L), malt extract (3 g/L), soy peptone (5 g/L), and glucose monohydrate (11 g/L), dissolved in 800 mL of distilled water.

Following the shake flask cultivation, 640 mL of the starter cultures were transferred into a 22-L fermenter (Biostat C, Braun Biotech Inc., Germany), with a total volume of 10 L of liquid cultivation medium. The defined bioreactor medium was prepared from separately sterilized stock solutions for glucose, phosphate, ammonium, salts, and trace elements, vitamins, and iron, which were combined with sterile water and preculture, according to established fed-batch protocols used at the Chair of Control, TU Berlin. The final medium composition is summarized in Table 1. Temperature and pH were maintained at 30 $^\circ$C and 6, respectively, using integrated PID controllers (Biostat C, Braun Biotech Inc., Germany) with pH measured with a glass electrode (405-DPAS-SC-K8S pH probe, Mettler-Toledo International Inc., USA) and controlled with 3 mol/L NaOH as the base ($u_{\textrm{B}}$) and 1 mol/L H$_2$SO$_4$ as the acid ($u_{\textrm{A}}$). The feeding rates of the fed-batch cultivations were realized by proportional-integral-controlled pump-scale systems and could be adjusted online via a custom-made process control system based on a central MySQL database [52]. P2000 (Fluka) was used as an antifoam agent. A Rushton turbine stirrer operating at 600 rpm was employed for agitation and the medium received continuous aeration at a rate of 15 standard liters per minute (slpm) of air. Throughout the fermentation process, glucose ($c_\textrm{Glc,Feed}=$ 450 g/L), ammonium ($c_\textrm{Am,Feed}=$ 30 g/L), and phosphate ($c_\textrm{Ph,Feed}=$ 24 g/L) were individually fed via a pump-scale system with the flow rates $u_{\textrm{Glc}}, u_{\textrm{Am}}, u_{\textrm{Ph}}$.

Substrate concentrations in the samples ($u_{\textrm{sample}}$) were determined photometrically using commercial enzyme kits such as glucose GOD FS (DiaSys, Germany), phosphate FS, and the Berthelot reaction for ammonium, following a fourfold determination in each case. The substrate concentrations were calculated from standard calibration curves according to the respective assay protocols. No dedicated spike-recovery experiments were performed for the enzymatic assays. For each sample, four absorbance values were recorded in a plate reader (Tecan, Switzerland) in 96-well microplates (Thermo Fisher Scientific, USA) and averaged for model calibration. Biomass was determined gravimetrically from duplicate filters by filtering a defined volume of the sample through a preweighed filter, rinsing with water, drying (24 h and 95 $^\circ$C), and measuring the cell dry weight. The dissolved oxygen tension (DOT) levels in the medium are monitored in real-time using an oxygen probe (InPro 6800, Mettler-Toledo International Inc., USA). All process data, including temperature, pH, DOT, feeding rates, and sample information, were continuously logged and stored in the MySQL database for subsequent analysis.

**Table 1:** Bioreactor medium composition for *S. cerevisiae* fed-batch cultivations

Component	Concentration
Glucose	15.0 g L$^{-1}$
KH$_2$PO$_4$	2.77 g L$^{-1}$
NH$_4$Cl	1.87 g L$^{-1}$
MgCl$_2\cdot$6 H$_2$O	0.698 g L$^{-1}$
KCl	1.35 g L$^{-1}$
CaCl$_2\cdot$2 H$_2$O	0.450 g L$^{-1}$
CuSO$_4\cdot$5 H$_2$O	3.51 mg L$^{-1}$
ZnSO$_4$	13.5 mg L$^{-1}$
MnSO$_4\cdot$H$_2$O	14.25 mg L$^{-1}$
Vitamin solution A/1/B6	28.5 mg L$^{-1}$
FeCl$_3\cdot$6 H$_2$O	23.4 mg L$^{-1}$
Glucose feed	450 g L$^{-1}$
Ammonium feed	30 g L$^{-1}$
Phosphate feed	24 g L$^{-1}$

Concentrations refer to the final 10 L working volume after combining separately sterilized stock solutions and preculture. KH$_2$PO$_4$ corresponds to approximately 1.93 g L$^{-1}$ phosphate, and NH$_4$Cl corresponds to approximately 0.63 g L$^{-1}$ ammonium

### In-line Raman spectroscopy

A tec5 MultiSpec^®^ process Raman spectrometer (tec5 AG, Steinbach, Germany) was used for in-line analysis. It was equipped with the following components

- Temperature-stabilized 785 nm diode laser with a power output of 500 mW;
- Peltier cooled 16-bit CCD diode array spectral sensor;
- Process Raman Probe 785 mini/MUB25 mm for Ingold socket.

The spectrometer covered the spectral range from 310 to 3190 cm^-1^ with a spectral resolution of 1 cm^-1^. The integration time per acquisition was 500 ms. The effective interval between stored spectra was approximately 5.5 s. The Raman probe was inserted into the SCHOTT ViewPort™ (SCHOTT AG, Landshut, Germany), which was autoclaved together with the vessel. No active anti-fouling strategy, such as mechanical wiping, probe-window coating, or automated cleaning, was applied. In our study severe probe-window fouling was not observed. Nevertheless, probe-window effects, bubble attachment, turbidity, and scattering are acknowledged as possible sources of spectral variation. Furthermore, no microscopic cell counting was performed for the Raman sampling volume. The in-line Raman signal is therefore interpreted as an ensemble measurement of a dense cell suspension, in which dissolved analytes, biomass-related scattering, cell-wall contributions, bubbles, turbidity, and possible probe-window effects may jointly contribute to the recorded spectra.

Data processing steps and subsequent data analysis were performed using MATLAB (R2022a, The MathWorks Inc., USA). Baseline correction was performed using adaptive iteratively reweighted Penalized Least Squares (airPLS) [54] with *λ * and degree of difference set to 63 and 1 recommended by [35]. The spectral regions, used for data analysis, featuring bands assigned by glucose, ammonium, phosphate, and amino acids assigned to biomass will be discussed in Section “Selection of wavenumbers”. Apart from airPLS baseline correction, no additional smoothing, scatter normalization, or derivative preprocessing was applied. This minimal preprocessing strategy was chosen to keep the comparison between CVQ, KS, UI, and RS interpretable and to isolate the effect of sample subset selection from additional preprocessing-model-selection decisions.

**Fig. 2:**
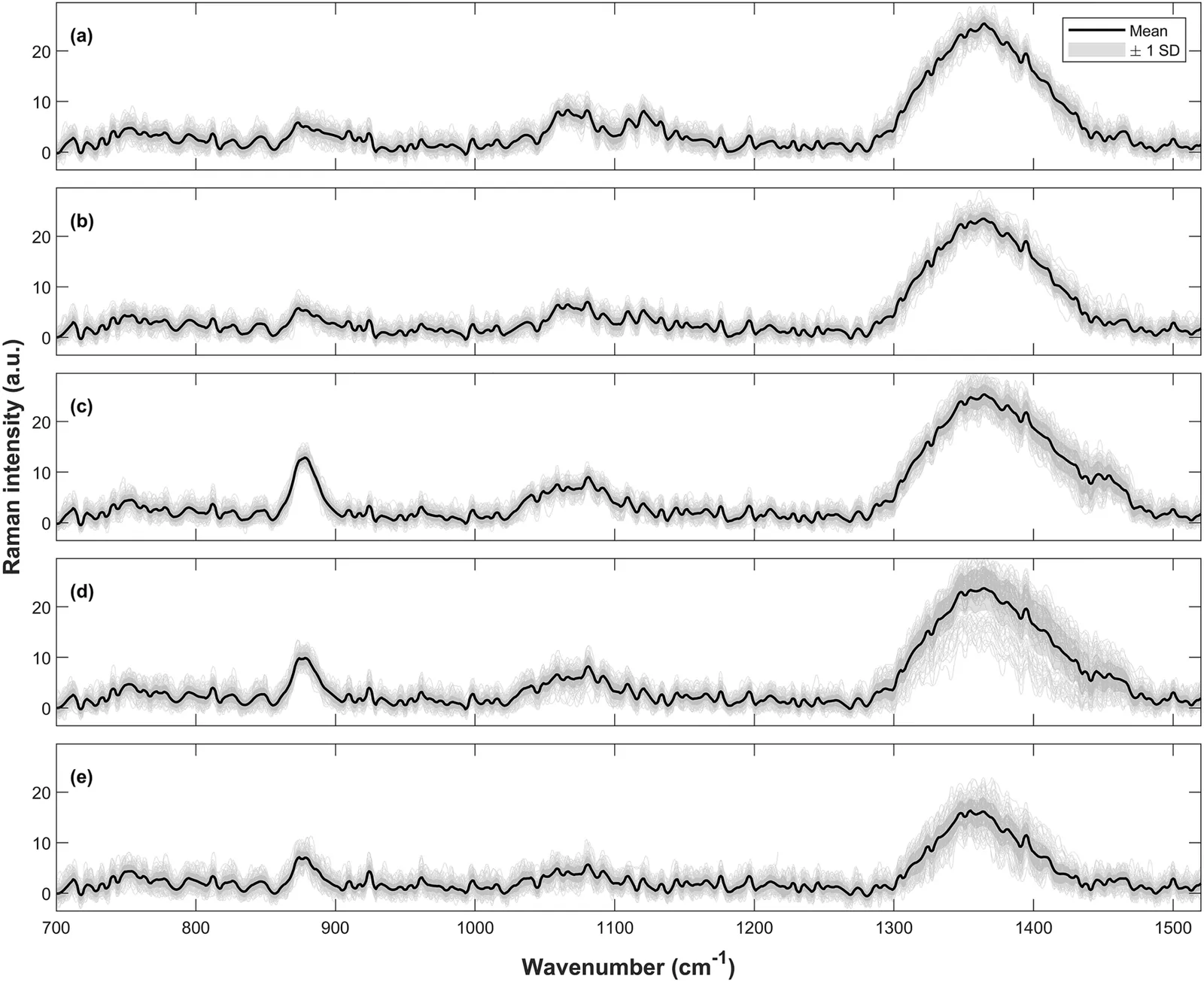
Representative airPLS-corrected in-line Raman spectra and variability across consecutive acquisitions from F1. Panels (**a**–**e**) correspond to candidate sampling indices 4, 6, 13, 19, and 22. Each panel shows 110 consecutive Raman acquisitions associated with the corresponding candidate offline sampling time. Thin grey lines represent the individual acquisitions, black lines denote the pointwise mean spectra, and grey envelopes indicate the mean ± one standard deviation calculated separately at each wavenumber. The spectra are displayed from 700 to 1520 cm$^{-1}$. The complete spectral overview of all sampling points is provided in Online Resource 1, Figs. S1–S4

Raman spectra were acquired continuously in-line in the fermentation broth throughout F1. In order to synchronize sample points in time with Raman spectra, the spectra were obtained during sampling, processing, and freezing. For each candidate offline sampling time, the first 110 consecutive stored Raman spectra after sample collection were selected. At an effective interval of approximately 5.5 s between stored spectra, these acquisitions covered approximately 10 min from the first to the last spectrum. The mean across the 110 acquisitions was calculated, resulting in one candidate spectrum per offline sampling time (Fig. 2 and Online Resource 1). The resulting 22 mean spectra constituted the spectral inputs used for CVQ- and KS-based sample subset selection. As an additional robustness check, the pointwise mean and median spectra were compared for each candidate sampling interval using an RMSE normalized by the peak-to-peak range of the corresponding mean spectrum.

### Raman-guided sample subset selection and baseline methods

The presented RGSS methods operate on the same pool of candidate sampling time points. Each candidate time point $t_{_\text {M},i}$ is represented by an averaged Raman spectrum $\mathbf{x}_i \in \mathbb {R}^{p}$, where *p* denotes the number of spectral variables used for selection. The overall aim is to select a subset $\mathcal {X}_{\text {sel}}$ from an initial set of unlabelled Raman spectra

1 $$\begin{aligned} \mathcal {X}_{\textrm{U}} = \{\mathbf{x}_i\}_{i=1}^{n_\textrm{t}}, \end{aligned}$$

where:

- The subscript “U” denotes the unlabelled (i.e., full) dataset,
- The subscript “sel” denotes the selected subset,
- $n_\text {t}$ represents the total number of spectra (sampling time points) collected during the experiment F1,
- $n_{\text {sel}}$ denotes the number of informative samples to be selected ($\mathcal {X}_{\text {sel}} \subset \mathcal {X}_\text {U}$, with $n_{\text {sel}} < n_\text {t}$).

The objective of sample subset selection is to choose $n_{\textrm{sel}} \in \{5,10\}$ candidate time points for subsequent offline reference analysis and nonlinear PI. The two subset sizes represent a strong reduction scenario (5 samples, 77% reduction) and a moderate reduction scenario (10 samples, 55% reduction), respectively. Furthermore, the initial model values at ($\textrm{BA}$ = 0) were used only for model initialization and were not included in the candidate set ($X_\textrm{U}$). Thus, ($n_\textrm{t}$ =22) refers exclusively to stored candidate offline samples that were linked to Raman spectra and considered for subset selection.

#### Pairwise spectral distance

The Euclidean distance is the common basis for the distance-based KS and kernel-based CVQ. Thus both methods use the same spectral information but apply different selection principles. We first define the pairwise Euclidean distance between two candidate spectra as

2 $$\begin{aligned} d_{ij}=\left\Vert \mathbf{x}_i-\mathbf{x}_j \right\Vert _2 . \end{aligned}$$

#### Kennard–Stone spectral subset selection

KS is widely used in chemometrics for calibration-set partitioning and representative sample subset selection from spectral data including NIR calibration studies and Raman-based bioprocess calibration transfer [41, 42, 55]. Following the classical KS algorithm [40], pairwise Euclidean distances (Eq. (2)) are computed between all candidate spectra. The first two spectra are the pair with the largest distance. Subsequently, KS iteratively adds the candidate spectrum that maximizes its minimum distance to the already selected subset.

#### Constrained vector quantization

Comprehensive derivations of the subset-constrained vector quantization technique (CVQ) employing mean discrepancy minimization are provided in [36–38]. Conceptually, CVQ is closely related to modern kernel-based methods that approximate a probability distribution by a small representative point set, minimizing kernel discrepancy measures such as maximum mean discrepancy [56, 57]. For an overview, the key points are briefly outlined below.

Krause et al. [38] applied CVQ on NIR spectra for the selection of training data in agricultural applications. The probability density of $\mathcal {X}_{\text {sel}}$ is obtained using the kernel trick [58] by minimizing an objective function over a binary selection vector $z \in \{0,1\}^{n_\textrm{t}}$, where $z_i = 1$ indicates inclusion of spectrum $x_i$ in $\mathcal {X}_{\text {sel}}$ and $z_i = 0$ indicates exclusion. This objective function, the mean discrepancy (MD), quantifies the similarity between the selected subset and the full dataset:

3 $$\begin{aligned} \text {MD}^2 = \frac{1}{n_{\text {sel}}^2} z^T \Phi z - \frac{2}{n_\text {t} n_{\text {sel}}} \mathbf{1}^T \Phi z, \end{aligned}$$

where $\mathbf{1} \in \mathbb {R}^{n_\text {t}}$ denotes an all-one vector and $\Phi \in \mathbb {R}^{n_\text {t} \times n_\text {t}}$ is the kernel matrix.

A radial basis function (RBF) kernel is employed to compute *Φ * as

4 $$\begin{aligned} (\Phi )_{ij} = \exp \left( - \frac{d_{ij}^2}{h} \right) , \end{aligned}$$

where the indices *i* and *j* denote individual spectra in the unlabelled dataset $\mathcal {X}_\text {U}$, incorporating the Euclidean distance Eq. (2).

The bandwidth parameter *h* is determined by

5 $$\begin{aligned} h = \det \left( \alpha _{\textrm{Scott}}^2 \Sigma \right) , \end{aligned}$$

where *Σ * is the covariance matrix of the dataset computed via Principal Component Analysis (PCA), and

6 $$\begin{aligned} \alpha _{\text {Scott}} = n_\text {t}^{-1/(d+4)} \end{aligned}$$

for $n_\text {t}$ spectra with dimensionality *d* (Krause et al. [38] refer to $\alpha _{\text {Scott}}$ as *Scott’s rule of thumb* [59]).

**Table 2:** Definition of variables used in constrained vector quantization (CVQ)

Symbol	Description
$n_\text {t}$	Total number of measured spectra (or samples)
$n_{\textrm{sel}}$	Total number of selected spectra (or samples) for offline analysis
$\mathbf{X}_U$	Set of $n_\text {t}$ unlabeled Raman spectra (measurement data)
$\mathbf{X}_\textrm{sel}$	Subset of $n_{\textrm{sel}}$ selected spectra (codebook)
*d*	Euclidean distance
$\mathbf{z}$	Binary indicator vector ($z_i = 1$ if $\mathbf{x}_i \in \mathbf{X}_\textrm{sel}$)
$MD^2$	Mean discrepancy objective value
$\mathbf {\Phi }$	Kernel matrix with entries $\Phi _{ij}$
*h*	Bandwidth parameter

The main variables and indices are summarized in Table 2. This greedy search algorithm iteratively minimizes the mean discrepancy (Eq. (3)) until $n_{\text {sel}}$ spectra with maximal informational value are selected.

#### Full-spectrum and wavenumber-selected variants

Both KS and CVQ are applied to the same Raman feature matrices. In the full-spectrum variants (FS-KS and FS-CVQ), the fingerprint region between 701 and 1520 cm$^{-1}$ is used and one common subset is selected for all four offline analytes. In the wavenumber-selected variants (WS-KS and WS-CVQ), analyte-specific spectral regions (Section “Selection of wavenumbers”) are used, resulting in separate selected subsets for the four analytes. This distinction is crucial for interpreting the analytical workload. In the present workflow, many physical samples can be collected and stored, whereas the major cost arises from the reference analysis of the chosen samples. Thus, WS-CVQ-05 and WS-KS-05 denote five selected reference assays per analyte, not necessarily five physical sampling time points in total. The union of physical sampling times can therefore be larger, but the number of costly reference values per analyte remains fixed. This analyte-specific temporal distribution allows the multi-output PI problem to use reference information from different process phases for different outputs.

#### Uniform-in-time and random sampling methods

To address whether RGSS provides a benefit beyond simple data reduction, two non-spectral baselines were added. Uniform-in-time (UI) sampling selected the available candidate samples closest to $n_{\textrm{sel}}$ equally spaced process time between the first and last candidate sampling point. Random sampling (RS) selected $n_{\textrm{sel}}$ candidates randomly and repeated for 20 fixed random seeds. The results are presented as median and the interquartile range (IQR). The subset sizes for UI and RS are $n_{\textrm{sel}}=10$ and $n_{\textrm{sel}}=5$.

#### Sample subset selection terminology

In this contribution, the total number of candidate offline sampling time points per analyte in F1 is $n_\text {t} = 22$. CVQ, KS, UI and RS are configured to select either $n_{\text {sel}} = 10$ (for FS-CVQ-10, FS-KS-10, WS-CVQ-10 and WS-KS-10, UI-10 and RS-10) or $n_{\text {sel}} = 5$ (for FS-CVQ-05, FS-KS-05, WS-CVQ-05, WS-KS-05, UI-05 and RS-05) informative spectra per analyte. All sample subset selection methods were subsequently evaluated using the same PI workflow and the same assessment criteria.

### Model description

The dynamic model is given by

7 $$\begin{aligned} \dot{\mathbf{x}}(t) = \mathbf{f}\left( \mathbf{x}(t),\boldsymbol{\theta },\mathbf{u}(t)\right) , \qquad \mathbf{y}(t) = \mathbf{h}\left( \mathbf{x}(t)\right) , \end{aligned}$$

with $\mathbf{x}(t)\in \mathbb {R}^{n_\text {x}}$, $\boldsymbol{\theta }\in \mathbb {R}^{n_\mathrm {\theta }}$, $\mathbf{u}(t)\in \mathbb {R}^{n_\text {u}}$, and $\mathbf{y}(t)\in \mathbb {R}^{n_\text {y}}$. The mechanistic model employed in this study describes the *S. cerevisiae* fermentation process and is based on ordinary differential equations (ODEs) described in [52, 53, 60, 61]. They are based on mass balances describing the dynamic behavior of key state variables cell dry weight (CDW), glucose (Glc), ammonium (Am), phosphate (Ph), dissolved oxygen tension (DOT) [61] in the medium, total volume of consumed base (B) [60], and the broth volume (V).

The model’s state variables and the corresponding ODEs are

8 $$\begin{aligned} \frac{\textrm{d}m_\textrm{CDW}}{\textrm{d}t}&= r_\textrm{CDW} \cdot m_\textrm{CDW} \end{aligned}$$

9 $$\begin{aligned} \frac{\textrm{d}m_\textrm{Glc}}{\textrm{d}t}&= -\left( Y_\textrm{Glc,CDW} \cdot r_\textrm{CDW} + r_\textrm{Maint}\right) \cdot m_\textrm{CDW} \nonumber \\&\quad + u_\textrm{Glc} \cdot c_\textrm{Glc,feed} \end{aligned}$$

10 $$\begin{aligned} \frac{\textrm{d}m_\textrm{Am}}{\textrm{d}t}&= -Y_\textrm{Am,CDW} \cdot r_\textrm{CDW} \cdot m_\textrm{CDW} + u_\textrm{Am} \cdot c_\textrm{Am,feed} \end{aligned}$$

11 $$\begin{aligned} \frac{\textrm{d}m_\textrm{Ph}}{\textrm{d}t}&= -Y_\textrm{Ph,CDW} \cdot r_\textrm{CDW} \cdot m_\textrm{CDW} + u_\textrm{Ph} \cdot c_\textrm{Ph,feed} \end{aligned}$$

12 $$\begin{aligned} \frac{\textrm{d}DOT}{\textrm{d}t}&= k_\textrm{L}a \cdot (DOT^* - DOT) \nonumber \\&\quad - \left( H \cdot Y_\textrm{O,Glc} \cdot r_\textrm{CDW} \cdot c_\textrm{CDW}\right) \end{aligned}$$

13 $$\begin{aligned} \frac{\textrm{d}m_\textrm{B}}{\textrm{d}t}&= Y_\textrm{B,Am} \cdot Y_\textrm{Am,CDW} \cdot r_\textrm{CDW} \cdot m_\textrm{CDW} \end{aligned}$$

14 $$\begin{aligned} \frac{\textrm{d}V}{\textrm{d}t}&= u_\textrm{Glc} + u_\textrm{Am} + u_\textrm{Ph} + u_\textrm{B} + u_\textrm{A} - u_\textrm{sample} \end{aligned}$$

and the state vector is defined as

$$\begin{aligned} \mathbf{x}^T = \left[ m_\textrm{CDW},\, m_\textrm{Glc},\, m_\textrm{Am},\, m_\textrm{Ph},\, DOT,\, m_\textrm{B},\, V \right] . \end{aligned}$$

Substrate limiting effects on aerobic biomass growth as well as maintenance are described using the expressions presented by Herold et al. [52] in which the automatic model building framework “advanced batch control" (ABC) was utilized:

$$\begin{aligned}&r_{\text {Glc}} = \mu _\textrm{Glc,max} \cdot \frac{{c_\textrm{Glc}}}{c_\textrm{Glc}+k_\textrm{m,Glc}} \cdot \frac{{c_\textrm{Am}}}{{c_\textrm{Am}+k_\textrm{m,Am}}} \cdot \frac{{c_\textrm{Ph}}}{{c_\textrm{Ph}+k_\textrm{m,Ph}}} \\&r_{\text {Maint}} = \mu _{\text {Maint,max}} \cdot \frac{{c_{\text {Glc}}}}{{c_{\text {Glc}}+k_\textrm{m,Maint}}} \end{aligned}$$

The ODE for DOT is stated in Eq. (12) and derived in [62]. The product of the two parameters, $k_\textrm{L}$ and *a*, is commonly employed as a single parameter to characterize a reactor’s oxygen transfer capacity. This parameter, denoted as $k_\textrm{L}a$ (h$^{-1}$), is referred to as the volumetric oxygen transfer coefficient and plays a crucial role in describing a bioreactor’s ability to transfer oxygen. $DOT^*$ represents the value of DOT in equilibrium with the gas bubble oxygen concentration and calibrated here as 100% air saturation. Furthermore, *H* is a constant derived from Henry’s law, which establishes a connection between the concentration of dissolved oxygen in g/L and % air saturation and is set to 14000 (% *· * L g^-1^) [61, 62].

The ODE for volume is represented in Eq. (14), which includes the sum of all inflows for Glc ($u_\textrm{Glc}$), Am ($u_\textrm{Am}$), Ph ($u_\textrm{Ph}$), base ($u_\textrm{B}$), acid ($u_\textrm{A}$) and sampling ($u_\textrm{sample}$)

$$\begin{aligned} \mathbf{u}^T = \left[ u_\textrm{Glc}, u_\textrm{Am}, u_\textrm{Ph}, u_\textrm{B}, u_\textrm{A},u_\textrm{sample}\right] . \end{aligned}$$

CDW, Glc, Am and Ph are measured as concentrations, so these measurement equations are composed of terms

$$\begin{aligned} c_i = \frac{m_i}{V}, \end{aligned}$$

where *V* represents the total volume, which is measured at-line. As stated in [53], the cumulative base volume is given by

$$\begin{aligned} m_\textrm{B} = \int _0^{t} u_\textrm{B} \, d\tau \end{aligned}$$

where $u_\textrm{B}$ is the volumetric base feed rate (L h$^{-1}$) and $m_\textrm{B}$ denotes the total volume of consumed base (L). Following the notation convention of [53], the symbol $m_\textrm{B}$ is retained for consistency with the cited model framework, despite representing a volume rather than a mass. The measurement vector consists of the terms

$$\begin{aligned} \mathbf{y}^T = \left[ c_\textrm{CDW}, c_\textrm{Glc}, c_\textrm{Am}, c_\textrm{Ph}, DOT, m_\textrm{B}\right] . \end{aligned}$$

For clarity and reproducibility, an extended list of all state variables, control inputs, and measured outputs of the mechanistic model together with their units and physical meaning is given in Table S1 in Online Resource 1.

When working with a new strain, it is advisable to research the strain’s origin and examine existing literature values to establish lower and upper bounds (LB and UB) for the model parameters $\boldsymbol{\mathbf {\theta }}$. For the given parameters,

$$\begin{aligned} \boldsymbol{\mathbf {\theta }} = \left[ \begin{array}{c} \mu _{\text {Glc}, \max } \\ \mu _{\text {Maint}, \max } \\ Y_{\text {Glc/CDW}} \\ Y_{\text {Am/CDW}} \\ Y_{\text {Ph/CDW}} \\ Y_{\text {O/Glc}} \\ Y_{\text {B/Am}} \\ k_\textrm{m,Glc} \\ k_\textrm{m,Maint} \\ k_\textrm{L}a \\ \end{array} \right] \end{aligned}$$

biologically plausible LB and UB could be as follows depending on fermentation conditions and strains:

- $\mu _\text {Glc,max}$: Biologically plausible LB and UB for the maximum specific growth rate utilizing glucose as a carbon source could range from approximately 0.1 to 0.5 1/h, based on typical values for *S. cerevisiae* [52, 60, 62].
- $\mu _{\text {Maint}, \max }$: The maximum specific maintenance rate could range from 0.01 to 0.2 1/h. LB and UB could be set around 0.005 1/h and 0.3 1/h.
- $Y_{\text {Glc/CDW}}$, $Y_{\text {Am/CDW}}$, $Y_{\text {Ph/CDW}}$: The aerobic consumption of glucose, ammonium, and phosphate on biomass production are represented by these yield coefficients. LB and UB could be set around 0.1 g/g and 20 g/g for $Y_{\text {Glc/CDW}}$, 0.01 and 2 g/g for $Y_{\text {Am/CDW}}$ and 0.01 and 2 g/g for $Y_{\text {Ph/CDW}}$ [62].
- $Y_{\text {O/Glc}}$: The oxygen consumption relative to glucose could range from 0.1 to 4 [62].
- $Y_{\text {B/Am}}$: The relationship between biomass production and ammonium consumption depends on various factors, including metabolic pathways and strain characteristics. LB and UB could range from 0.01 to 0.5, depending on specific conditions and the organism under consideration.
- $k_\textrm{m,Glc}, k_\textrm{m,Maint}$: The monod constants for substrate concentrations regarding growth and maintenance can vary from 0.01 to 10 g/L. LB and UB for these parameters could be set around 0.001 g/L and 20 g/L. $k_\textrm{m,Am}$ and $k_\textrm{m,Ph}$ are set constant as in [52] and are therefore not optimized in the PI.
- $k_\textrm{L}a$: The volumetric oxygen transfer coefficient depends on the type of bioreactor and operating conditions. Typical values for bioreactors could range from 25 to 300 1/h. LB and UB could be set around 20 1/h and 1000 1/h.

Please note that these bounds are based on typical values for *S. cerevisiae* and should be adjusted to suit specific experimental conditions and the strain under investigation.

**Table 3:** Model parameters $\boldsymbol{\theta }$ with units, biological meaning, and assumed lower (LB) and upper bounds (UB) for *S. cerevisiae*

Parameter	Unit	LB	UB	Description
$\mu _{\text {Glc},\max }$	h$^{-1}$	0.10	0.50	Maximum specific growth rate on glucose
$\mu _{\text {Maint},\max }$	h$^{-1}$	0.005	0.30	Maximum specific maintenance rate
$Y_{\text {Glc/CDW}}$	g g$^{-1}$	0.10	20.0	Glucose consumption per biomass produced
$Y_{\text {Am/CDW}}$	g g$^{-1}$	0.01	2.0	Ammonium consumption per biomass produced
$Y_{\text {Ph/CDW}}$	g g$^{-1}$	0.01	2.0	Phosphate consumption per biomass produced
$Y_{\text {O/Glc}}$	g g$^{-1}$	0.10	4.00	Oxygen consumed per glucose utilized
$Y_{\text {B/Am}}$	g g$^{-1}$	0.01	0.50	Base formation per ammonium consumed
$k_{\textrm{m,Glc}}$	g L$^{-1}$	0.001	20.0	Monod constant for glucose uptake
$k_{\textrm{m,Maint}}$	g L$^{-1}$	0.001	20.0	Monod constant for maintenance metabolism
$k_\textrm{L}a$	h$^{-1}$	20	1000	Volumetric oxygen transfer coefficient

Bounds are based on literature values and may be adjusted for other strains or process conditions

Table 3 summarizes all parameters of the mechanistic model, their physical units, biological meaning, and the lower and upper bounds (LB, UB) used during PI. The ranges reflect biologically plausible values for *S. cerevisiae* reported in the literature [52, 60, 62].

### Parameter identification

PI was performed to fit the output of the model $\mathbf{y}^\textrm{model}$ to measured values $\mathbf{y}^\textrm{M}$. Model predictions were calculated by integrating the model equation with an ODE solver (*ode*15*s*) by matlab as a function of the model parameters $\boldsymbol{\mathbf {\theta }}$ and inputs $\mathbf{u}$. Initial conditions, which are also to be determined, are included as additional parameters in $\boldsymbol{\mathbf {\theta }}$ and will not be listed separately in subsequent discussions. They are not part of the RGSS candidate sample set. A cost function for the PI was formulated as a modified *maximum-likelihood-estimation* [16] for $n_\textrm{t}$ points in time and $n_\textrm{m}$ measurement variables:

15 $$\begin{aligned}&\Phi _\textrm{PI} = \sum _{k=1}^{n_\textrm{t}} \sum _{l=1}^{n_\textrm{m}} \left( \mathbf{y}_{k,l}^\textrm{M} - \mathbf{y}_{k,l}^\textrm{model}(t,\mathbf{u},\mathbf {\theta })\right) ^T W_{k,l} \tilde{C}_{k,l}^{-1} \left( \mathbf{y}_{k,l}^\textrm{M}\right. \nonumber \\&\left. \quad - \mathbf{y}_{k,l}^\textrm{model}(t,\mathbf{u},\boldsymbol{\mathbf {\theta }})\right) . \end{aligned}$$

$\tilde{C}_{k,l}^{-1}$ is the inverse of the measurement covariance matrix of the offline and online reference data. Heine et al. [63] proposed an additional weighting of the measurements with a weight $W_{k,l}$ to account for the influence of frequently measured variables compared to rarely measured ones. This weight can be used as well, for example, when only few or no manual samples are taken during nighttime, but one or two at the end of a fermentation the next morning. For a human modeler, these measurements often contain important information, but a nonlinear optimization method exploiting the standard maximum likelihood-estimation cannot judge their importance correctly. Furthermore, for each RGSS, the number of available measurement points in time may differ between offline and online variables. While RGSS reduces the number of costly offline sampling points in time compared to $n_{\textrm{sel}}$, the online-accessible variables (e.g., *DOT* and $m_\textrm{B}$) are evaluated on the full measurement grid.

The basis for the PI is a mathematical optimization that changes the parameter $\mathbf {\theta }$ values in order to minimize criterion $\Phi _\textrm{PI}$ (Eq. (15)):

$$\begin{aligned} \hat{\mathbf {\boldsymbol{\theta }}}&=\arg \underset{\mathbf {\theta }}{\operatorname {min}} \Phi _\textrm{PI} \\ \text{ s.t.: } \dot{\mathbf{x}}&=\mathbf{f}(\mathbf{x}(t), \mathbf{u}(t), \mathbf {\boldsymbol{\theta }}), \quad \mathbf{x}\left( t_{0}\right) =\mathbf{x}_{0} \\ \mathbf {\boldsymbol{\theta }}_\textrm{LB}&\le \mathbf {\boldsymbol{\theta }} \le \mathbf {\boldsymbol{\theta }}_\textrm{UB} , \end{aligned}$$

where $\mathbf{f}$ is the right-hand side of the ODEs that define the biological process model. The parameter values $\boldsymbol{\mathbf {\theta }}$ are constrained by $\boldsymbol{\mathbf {\theta }}_\textrm{LB}$ and $\boldsymbol{\mathbf {\theta }}_\textrm{UB}$, as described in Table 3 above.

To ensure that the nonlinear optimization does not converge to local optima, multiple optimizations with various initial conditions were conducted and combined with a global metaheuristic. The optimization variable space is listed as LB and UB and is given in Section “Model description”. By utilizing Latin hypercube sampling (LHS) [46, 47], optimization variables or samples can be drawn from a space of random variables, which is divided into $n_\textrm{LHS}$ equally probable parts according to the probability distribution, and a random sample is drawn from each of these parts. Initial parameter values were generated for identification using LHS, which strategically selects diverse starting points within the constrained parameter space, ensuring comprehensive coverage. This process entails 1000 LHS iterations and 10 random starts, totaling 10,000 PIs. From these outcomes, the top 100 performers to serve as parents in the genetic algorithm were selected. Within this algorithm, new parameter combinations (children) were derived from the parent set through exchanges and slight perturbations (mutations). These children were then evaluated using a fitness function (see (15)), ultimately identifying the optimal parameter set among the 10,000 PIs.

A PI metaheuristic strategy presented in [45], combining LHS-based multi-start initialisation, a GA with gradient-based local refinement, and identifiability-guided bound tightening, was applied to the entire dataset F1, and subsequently to datasets with reduced samplings obtained via the CVQ-based RGSS strategy. To cross-validate the accuracy of the estimation, the normalized root mean squared error (NRMSE) between estimated $\mathbf{y}^\textrm{model}$ and measurement values $\mathbf{y}^\textrm{M}$ of F2 is utilized. The NRMSE is calculated as follows:

16 $$\begin{aligned} \textrm{NRMSE} = \frac{\left\| \mathbf{y}^\textrm{model} - \mathbf{y}^\textrm{M} \right\| _2}{\left\| \textrm{mean}(\mathbf{y}^\textrm{M}) - \mathbf{y}^\textrm{M} \right\| _2}. \end{aligned}$$

In the context of PI and cross-validation (CV), we refer to this criterion as NRMSE-AV (for auto-validation) and NRMSE-CV (for cross-validation). While other normalization approaches (e.g., range-based or standard-deviation-based) could in principle be used, we chose NRMSE because it is dimensionless, directly comparable across analytes with very different units and dynamic ranges (e.g. DOT and $c_\textrm{Am}$), and widely adopted in the bioprocess modeling literature [8, 21, 43, 53].

Furthermore, the volume (V) is not included in the PI because it is merely a sum of known inputs and is therefore known. Moreover, it does not contain any information regarding the parameters.

### Practical identifiability analysis

Considering a model as in Eq. (7), parameter identifiability is assessed using the sensitivity-based parameter subset selection (SbS) method [48, 49]. Practical identifiability has multiple methodological interpretations in the literature, which are sensitivity-matrix-based classifications (as used here) versus confidence-interval-based or profile-likelihood-based approaches [64]. Our analysis follows the sensitivity-based framework in which parameters are classified as practically identifiable if their sensitivities are well-conditioned and numerically distinguishable from the available measurement data [48].

This approach is based on the sensitivity of the model outputs $\mathbf{y}(t)$ with respect to the parameters $\boldsymbol{\theta } \in \mathbb {R}^{n_\theta }$:

17 $$\begin{aligned} \mathbf{S}_\text {y}(t) = \frac{\partial \mathbf{y}(t)}{\partial \boldsymbol{\theta }} \in \mathbb {R}^{n_\text {y} \times n_\theta }, \end{aligned}$$

which is calculated via the forward sensitivity approach. In this method, the sensitivity of the states $\mathbf{x}(t)$ with respect to parameters is first computed by integrating the sensitivity ODE

18 $$\begin{aligned} \dot{\mathbf{S}}_\text {x}(t) = \frac{\partial \mathbf{f}}{\partial \mathbf{x}}\,\mathbf{S}_\text {x}(t) + \frac{\partial \mathbf{f}}{\partial \boldsymbol{\theta }}, \end{aligned}$$

together with the state model and the initial condition $\mathbf{S}_x(t_0) = \partial \mathbf{x}(t_0)/\partial \boldsymbol{\theta }$. The corresponding output sensitivities are then evaluated as

19 $$\begin{aligned} \mathbf{S}_\text {y}(t_k) = \frac{\partial \mathbf{y}(t_k)}{\partial \boldsymbol{\theta }} = \frac{\partial \mathbf{h}}{\partial \mathbf{x}}\,\mathbf{S}_\text {x}(t_k) + \frac{\partial \mathbf{h}}{\partial \boldsymbol{\theta }}, \end{aligned}$$

where $\{t_k\}_{k=1}^{N_{\text {meas}}}$ denote the measurement time points used for the respective sampling strategy, and $N_{\text {meas}}$ is the total number of available measurements for the considered outputs.

To account for measurement uncertainty in the identifiability analysis, the output sensitivities are scaled with the measurement covariance matrix $\tilde{C}$:

20 $$\begin{aligned} \tilde{\mathbf{S}}_\text {y}(t_k) = \tilde{C}^{-1/2} \mathbf{S}_\text {y}(t_k). \end{aligned}$$

This scaling weights sensitivities by measurement uncertainty and accounts for error propagation from experimental data to parameter estimates, ensuring that practical identifiability reflects both model structure and data quality. The scaled sensitivities of all discrete measurements are stacked into

21 $$\begin{aligned} \tilde{\mathbf{S}} = \begin{bmatrix} \tilde{\mathbf{S}}(t_1) \\ \vdots \\ \tilde{\mathbf{S}}\left( t_{N_\textrm{meas}}\right) \end{bmatrix} \in \mathbb {R}^{(N_\text {meas} n_\text {y}) \times n_\theta }, \end{aligned}$$

where $n_\text {y}$ is the number of outputs. A rank-revealing singular value decomposition (SVD) of $\tilde{\mathbf{S}}$ is computed:

$$\begin{aligned} \tilde{\mathbf{S}} = \sum _{i=1}^{n_\theta } \boldsymbol{\mu }_i \,\varsigma _i\, \mathbf{v}_i^\top , \end{aligned}$$

with ordered singular values $\varsigma _1 \ge \varsigma _2 \ge \cdots \ge \varsigma _{n_\theta } \ge 0$.

Following [48], the numerical *\varepsilon *-rank $r_\varepsilon$ of $\tilde{\mathbf{S}}$ is defined as the maximum number of singular values for which the sub-condition number

22 $$\begin{aligned} \kappa _i = \frac{\varsigma _1}{\varsigma _i} \end{aligned}$$

and the sub-collinearity index

23 $$\begin{aligned} \gamma _i = \frac{1}{\varsigma _i} \end{aligned}$$

remain below critical thresholds. The empirical bounds $\kappa _{\max } = 1000$ and $\gamma _{\max } = 10^{10} \dots 10^{15}$ are adopted here. The condition number bound $\kappa _{\max }$ ensures numerical stability, meaning each singular value $\varsigma _i$ is at most 1000 times smaller than the largest one ($\varsigma _1$). The collinearity bound $\gamma _{\max }$ controls near-linear dependencies between parameter sensitivities.

Well-conditioned singular values lie above the lower bound given by the *\varepsilon *-threshold:

24 $$\begin{aligned} \varepsilon = \max \left\{ \varepsilon _\kappa = \frac{\varsigma _1}{\kappa _{\max }}, \quad \varepsilon _\gamma = \frac{1}{\gamma _{\max }} \right\} . \end{aligned}$$

Parameters associated with singular values violating these thresholds are classified as practically non-identifiable. Thus, a subset of practically identifiable parameters is selected.

### Implementation

All algorithms were implemented in MATLAB 2022b on an Intel(R) Core(TM) i9-10920X CPU 3.5GHz, with parallelization utilizing 12 cores. The distribution of every PI on multiple cores using parallelized for-loops *parfor* resulted in a 10-fold increase in optimization speed. The PI optimizations were conducted utilizing the *fmincon* and *ga*. Global observability and structural identifiability was analytically verified through Lie derivatives employing the STRIKE-GOLDD framework [65], developed by Villaverde et al. [66, 67].

All subset-selection strategies (FD, CVQ, KS, UI and RS) were evaluated with identical PI settings, a fixed random seed and the same precomputed LHS initialization, in order to make the optimization steps reproducible and avoid differences between sampling strategies. RS had additionally multiple fixed random seeds to quantify variability due to the stochastic sample selection itself. Compact MATLAB reference implementations of the CVQ- and KS-based spectral subset-selection procedures are provided in Online Resource 1 (Listings 1–2). The complete selected time points for deterministic and RS scenarios are provided as supplementary tables.

### Selection of wavenumbers

The CVQ method as applied by [38] utilizes the entire Raman spectrum for identifying sample subsets (FS-CVQ) with high information content. Nevertheless, it is more efficient to incorporate information about known molecules into the selection of wavenumbers (WS-CVQ), thereby reducing the search or variable space specific to the molecule for analyte-specific subset selection. Yang et al. [35] reported that full-spectrum prediction may yield poor accuracy and focused on the Raman fingerprint range between 701 and 1520 $\text {cm}^{-1}$, based on Virtanen et al. [68]. In the present study, all literature-derived bands selected for biomass-related cell-wall components, glucose, ammonium, and phosphate are located within this fingerprint region. Therefore, the spectral region for FS-CVQ and FS-KS were defined between (701–1520 $\text {cm}^{-1}$). Higher spectral regions above 1520 cm$^{-1}$ were not systematically evaluated in the present benchmark and remain a direction for future work. Alternative automated wavenumber-selection strategies were not evaluated in the present study. Such strategies form a broad research field, ranging from classical variable-selection and sparsity-based approaches to model-based feature importance and explainable-AI methods for Raman spectroscopy [28, 69]. Combining RGSS with automated wavenumber-selection and preprocessing optimization is therefore considered an important extension for future work.

In general, the yeast cell wall is primarily composed of mannoproteins (30–40%), 1,6-*β *-glucan (5–10%), 1,3-*β *-glucan (30–45%) and chitin (1.5–6%) [70–73]. Mannoproteins are proteoglycans composed of approximately 20% protein and 80% d-mannose [74]. Wan et al. [75] conducted a detailed study of mannoproteins or more specifically on amino acids compositions of the yeast cell wall. This analysis revealed that the most abundant amino acids are aspartic acid (12.12%) and glutamic acid (12.91%), followed by serine and phenylalanine. Since *β *-glucan consists solely of d-glucose linked by *β *-glycosidic bonds, and we also aim to extract information on glucose as a dissolved medium from the spectra, we will exclusively utilize the fingerprints of amino acids for biomass tracking to prevent inadvertent confusion with dissolved glucose. Additionally, we utilized findings from publications detailing the fingerprints peaks of yeast biomass itself [33, 72, 73, 76–80]. Pontius et al. [77] detailed the Raman peaks related to phosphate and ammonium in aqueous solution. These selected wavenumbers for specific reference data are outlined in Table 4. Please note that these wavenumbers were not used as single values for the WS-CVQ, but rather as a range around the specified values (– 5 and + 5 $\text {cm}^{-1}$), to compensate for noise and peak shifts while still retaining information from the spectra.

Figure 2 illustrates the individual baseline-corrected Raman acquisitions and corresponding mean spectra for five representative candidate sampling intervals for F1. No additional outlier-removal was applied. Furthermore, the literature-derived WS feature regions are visualized together with all 22 mean candidate spectra in Online Resource 1, Fig. S1. Grey bands denote biomass-related regions, red denotes the glucose-related region, green denotes the ammonium-related region, and blue denotes phosphate-related regions. Additionally, the Raman acquisitions of the remaining sampling points are illustrated in Online Resource 1, Figs. S2-S4.

**Table 4:** Literature-derived [33, 71–73, 76–80] Raman bands used for wavelength selection in WS-CVQ

Reference data	Fingerprint peaks (cm$^{-1}$)	Source(s)
*Biomass-related bands*		
Yeast surface	724, 793, 959, 1003, 1030 1083, 1126, 1204, 1245 1337, 1362, 1452	[71, 72, 76, 80]
Aspartic acid	938, 1335, 1421	[79]
Glutamic acid	868, 1311, 1409	[79]
Serine	852, 1009, 1129 1325, 1462	[79]
Phenylalanine	834, 1003, 1032, 1219	[79]
*Medium/dissolved components*		
Glucose (aq.)	874, 1056, 1080, 1118 1123, 1360, 1500	[33, 72, 73, 76–78]
Ammonium (aq.)	1455	[77]
Phosphate (aq.)	987, 1076, 1157	[77]

## Results

In this section, AV and CV results are assessed via NRMSE for the full-data reference and all sample subset-selection strategies. For the full-data reference and the CVQ-based RGSS scenarios, an additional practical identifiability analysis based on SbS is performed. Figure 2 provides a direct visualization of the Raman spectra used to construct the candidate spectra. The comparison between mean and median aggregation showed close agreement across the 22 candidate sampling intervals. The median range-normalized RMSE between the mean and median spectra was 0.00574, and the maximum was 0.02675, corresponding to 0.574% and 2.675% of the respective peak-to-peak spectral range. The mean spectra were therefore used as inputs for CVQ and KS. The complete interval-wise comparison is provided in Online Resource 1, Table S6. Furthermore, Online Resource 1, Fig. S1 illustrates the representative intervals in the context of all 22 mean candidate spectra, and Figs. S2–S4 show the individual acquisitions for the remaining candidate sampling intervals.

**Fig. 3:**
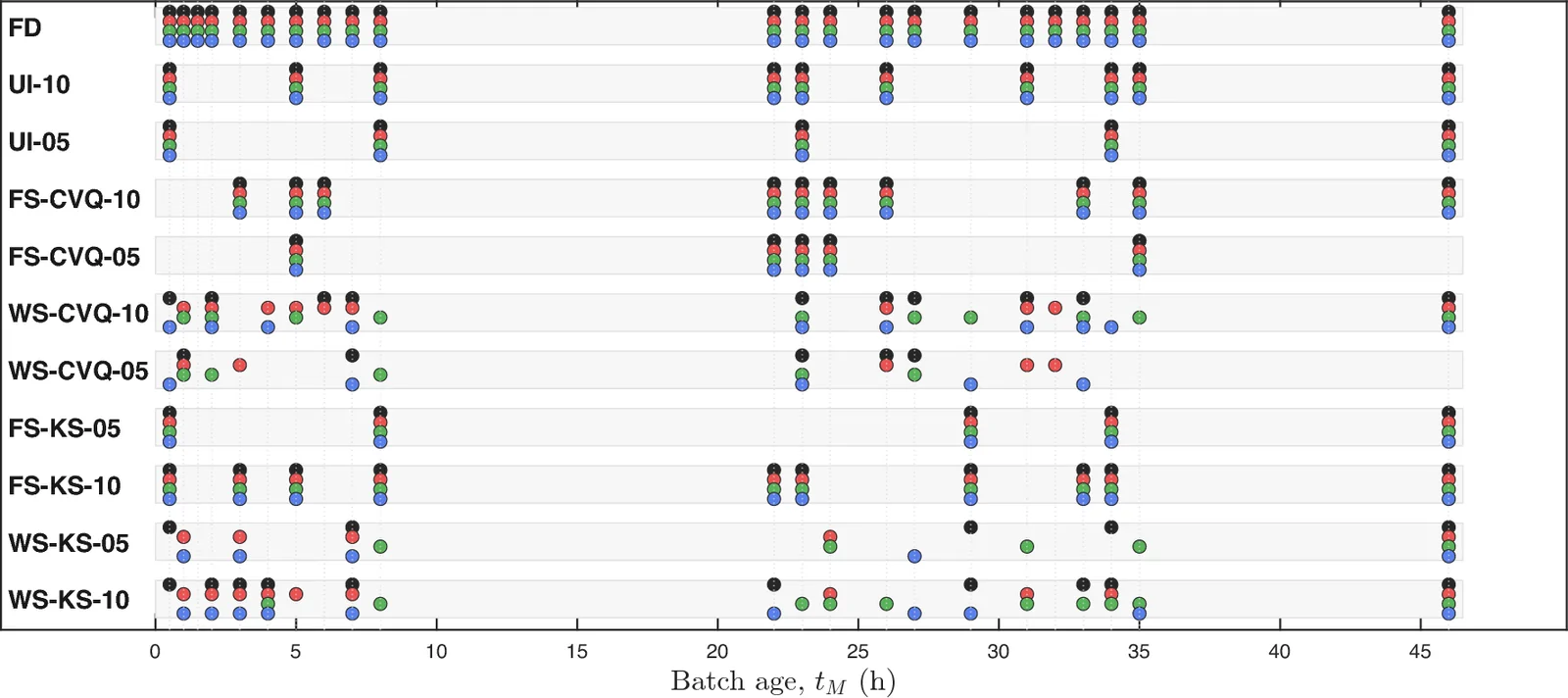
Offline sampling time points selected by the sample subset-selection strategies on F1. Each row within a method block corresponds to one analyte, with marker colors denoting CDW (black), Glc (red), Am (green), and Ph (blue). Full-data (FD) uses all 22 candidate offline samples, UI distributes samples only according to time, whereas FS-based strategies assign one common subset to all analytes. In contrast, WS-based strategies select analyte-specific subsets, which leads to a broader overall temporal coverage while maintaining the same number of reference assays per analyte. Initial values at ($\textrm{BA}$=0) are not part of the 22 candidate offline samples and are therefore not considered for subset selection. RS is not shown because 20 stochastic realizations were evaluated and are summarized separately in the Online Resource 1

The selected time points for each sample subset-selection method are depicted in Fig. 3. The primary performance metric for the baseline comparison is the summed NRMSE of the offline analytes, $\sum \textrm{NRMSE}_{\textrm{off}}$, because the subset-selection strategies target the reduction of costly offline reference assays. Consequently, these core metrics are highlighted in bold within the respective tables. Online and all-output NRMSE values are additionally reported as consistency metrics for the identified process model.

Online Resource 1 provides the complete Raman spectral documentation and additional PI results. Figure S1 shows all 22 mean candidate spectra, whereas Figs. S2–S4 show the individual acquisitions for the candidate sampling intervals not included in Fig. 2. Additional AV and CV simulation plots for FD, FS-CVQ, and WS-CVQ are provided in Figs. S5–S13. Online Resource 1 further contains an extended overview of model variables and units (Table S1), deterministic selected sampling times per analyte and their physical-time unions (Tables S2–S3), the complete RS-05 and RS-10 sampling realizations (Tables S4–S5), and the mean-versus-median robustness assessment for Raman acquisitions (Table S6). Compact reference implementations of CVQ and KS are provided in Listings 1–2.

### Auto-validation

PI was performed on dataset F1 using the full-data reference (FD), the CVQ-based RGSS scenarios, the KS spectral baselines, and the UI and RS non-spectral baselines. The quality of the PI was quantified by the NRMSE-AV value calculated according to Eq. (16). For all reduced strategies, NRMSE-AV was evaluated on all available F1 measurements rather than only on the subset used during PI. This provides a common AV grid and assesses how well PI based on reduced data predicts measurements that were not directly used during PI.

**Fig. 4:**
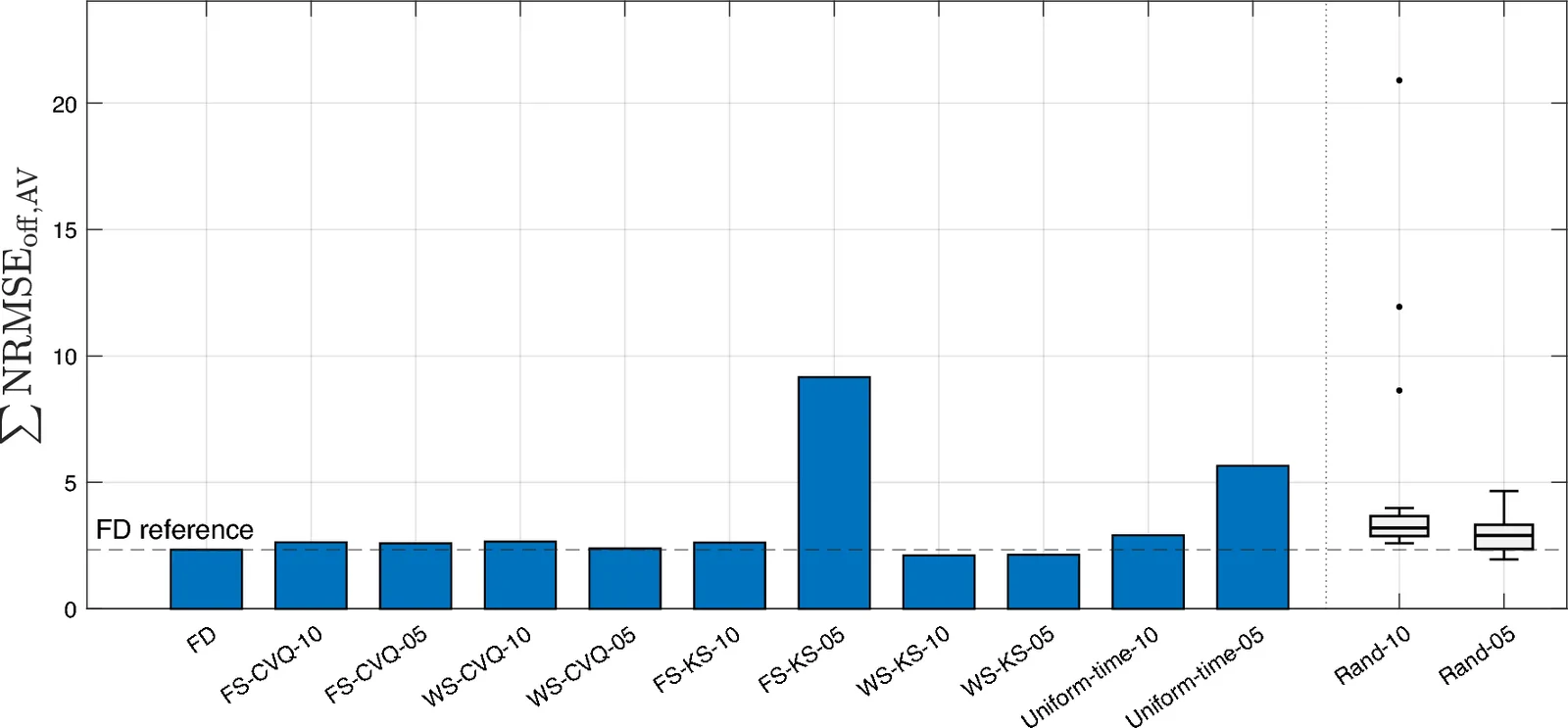
Auto-validation (AV) benchmark of reduced sampling strategies based on the summed NRMSE of the four offline analytes on F1 with full data. Deterministic strategies are shown as single values, whereas RS is shown as boxplots over 20 stochastic realizations. The full-data reference (FD) is shown as dashed reference line

Figure 4 summarizes the AV benchmark using $\sum \textrm{NRMSE}_{\textrm{off,AV}}$. Detailed analyte-wise values for the original FD and CVQ scenarios are depicted in Table 5 while additional KS, UI, and RS baselines are reported separately in Table 6 and Table 7. FD yields $\sum \textrm{NRMSE}_{\textrm{off,AV}}=2.34$. WS-CVQ-05 remains close to this value (2.39) and WS-KS-05 and WS-KS-10 are similarly low (2.14 and 2.12). FS-KS-05 and Uniform-time-05 show larger AV errors (9.16 and 5.65), indicating that not all reduced subsets are equally informative even on F1. PI with RS performs comparatively well in AV, with median and IQR values of 2.90 [2.39–3.30] for RS-05 and 3.20 [2.94–3.6] for RS-10.

Increasing the random subset size from five to ten did not improve the median AV performance in this benchmark. The median $\sum \textrm{NRMSE}_{\textrm{off,AV}}$ increased slightly from 2.90 for RS-05 to 3.20 for RS-10. Because the RS-05 and RS-10 realizations were generated independently, this comparison does not represent the sequential addition of five samples to an otherwise fixed subset.

In addition to the offline analytes, the online variables (DOT) and ($m_\textrm{B}$) were evaluated to assess whether PI based on reduced offline subsets remains consistent with the continuously available process measurements. For the CVQ scenarios, the full-data reference yielded the lowest all-output AV error ($\sum \textrm{NRMSE}_{\textrm{all,AV}}$=3.51), followed by WS-CVQ-05 (3.66), FS-CVQ-05 (3.83), WS-CVQ-10 (3.95), and FS-CVQ-10 (4.12). Among the baselines, WS-KS-05 and WS-KS-10 showed low all-output AV errors of 4.47 and 4.62, whereas FS-KS-05 was clearly less favourable (14.84), mainly due to the large error in ($m_\textrm{B}$). The RS baselines yielded median ($\sum \textrm{NRMSE}_{\textrm{all,AV}}$) values of 5.81 [4.97–6.71] for RS-05 and 6.07 [5.60–7.47] for RS-10.

**Table 5:** NRMSE-AV results for four offline analytes and two online measurements, dissolved oxygen tension (DOT) and cumulative base volume ($m_\textrm{B}$) under five sampling strategies

Analyte	FD	FS-CVQ-10	FS-CVQ-05	WS-CVQ-10	WS-CVQ-05
*Offline analytes (NRMSE-AV)*					
$c_\textrm{CDW}$	0.43	0.41	0.50	0.43	0.40
$c_\textrm{Glc}$	0.30	0.26	0.26	0.32	0.26
$c_\textrm{Am}$	0.30	0.23	0.44	0.33	0.44
$c_\textrm{Ph}$	1.31	1.73	1.39	1.58	1.30
$\sum (\mathrm {NRMSE_{off}})$	**2**.**34**	**2**.**63**	**2**.**59**	**2**.**66**	**2**.**39**
*Online measurements (NRMSE-AV)*					
*DOT*	1.00	1.32	1.06	1.11	1.08
$m_\textrm{B}$	0.17	0.17	0.18	0.18	0.19
$\sum (\mathrm {NRMSE_{all}})$	**3**.**51**	**4**.**12**	**3**.**83**	**3**.**95**	**3**.**66**

Shown in bold are the summed offline NRMSE values $\sum (\mathrm {NRMSE_{off}})$, and the total $\sum (\mathrm {NRMSE_{all}})$

**Table 6:** NRMSE-AV for four offline analytes and two online measurements, dissolved oxygen tension (DOT) and cumulative base volume ($m_\textrm{B}$) under KS and UI baseline strategies

Analyte	FS-KS-10	FS-KS-05	WS-KS-10	WS-KS-05	UI-10	UI-05
*Offline analytes (NRMSE-AV)*						
$c_\textrm{CDW}$	0.32	1.43	0.19	0.53	0.41	0.95
$c_\textrm{Glc}$	0.42	1.08	0.62	0.71	1.25	0.58
$c_\textrm{Am}$	1.13	1.10	0.60	0.22	0.51	0.22
$c_\textrm{Ph}$	0.75	5.56	0.70	0.69	0.74	3.90
$\sum (\mathrm {NRMSE_{off}})$	**2**.**62**	**9**.**16**	**2**.**12**	**2**.**14**	**2**.**91**	**5**.**65**
*Online measurements (NRMSE-AV)*						
*DOT*	2.61	2.30	2.33	2.32	4.05	2.01
$m_\textrm{B}$	0.38	3.38	0.18	0.01	0.09	0.14
$\sum (\mathrm {NRMSE_{all}})$	**5**.**62**	**14**.**84**	**4**.**62**	**4**.**47**	**7**.**05**	**7**.**80**

Shown in bold are the summed offline NRMSE values $\sum (\mathrm {NRMSE_{off}})$, and the total $\sum (\mathrm {NRMSE_{all}})$

**Table 7:** NRMSE-AV for four offline analytes and two online measurements, dissolved oxygen tension (DOT) and cumulative base volume ($m_\textrm{B}$) under RS-10 and RS-05 sampling strategies

Analyte	RS-10	IQR	RS-05	IQR
*Offline analytes (NRMSE-AV)*				
$c_\textrm{CDW}$	0.38	[0.36–0.43]	0.37	[0.34–0.40]
$c_\textrm{Glc}$	0.72	[0.65–0.91]	0.69	[0.62–0.80]
$c_\textrm{Am}$	1.24	[0.96–1.34]	0.95	[0.44–1.28]
$c_\textrm{Ph}$	0.95	[0.73–1.13]	0.76	[0.70–1.06]
$\sum (\mathrm {NRMSE_{off}})$	**3**.**20**	**[2.94–3.65]**	**2**.**90**	**[2.39–3.30]**
*Online measurements (NRMSE-AV)*				
*DOT*	2.54	[2.43–2.57]	2.49	[2.37–2.53]
$m_\textrm{B}$	0.10	[0.04–0.31]	0.10	[0.04–0.52]
$\sum (\mathrm {NRMSE_{all}})$	**6**.**07**	**[5.60–7.47]**	**5**.**81**	**[4.97–6.71]**

Values are reported as median and interquartile range (IQR) over 20 random runs.Shown in bold are the summed offline NRMSE values $\sum (\mathrm {NRMSE_{off}})$, and the total $\sum (\mathrm {NRMSE_{all}})$

### Cross-validation

**Fig. 5:**
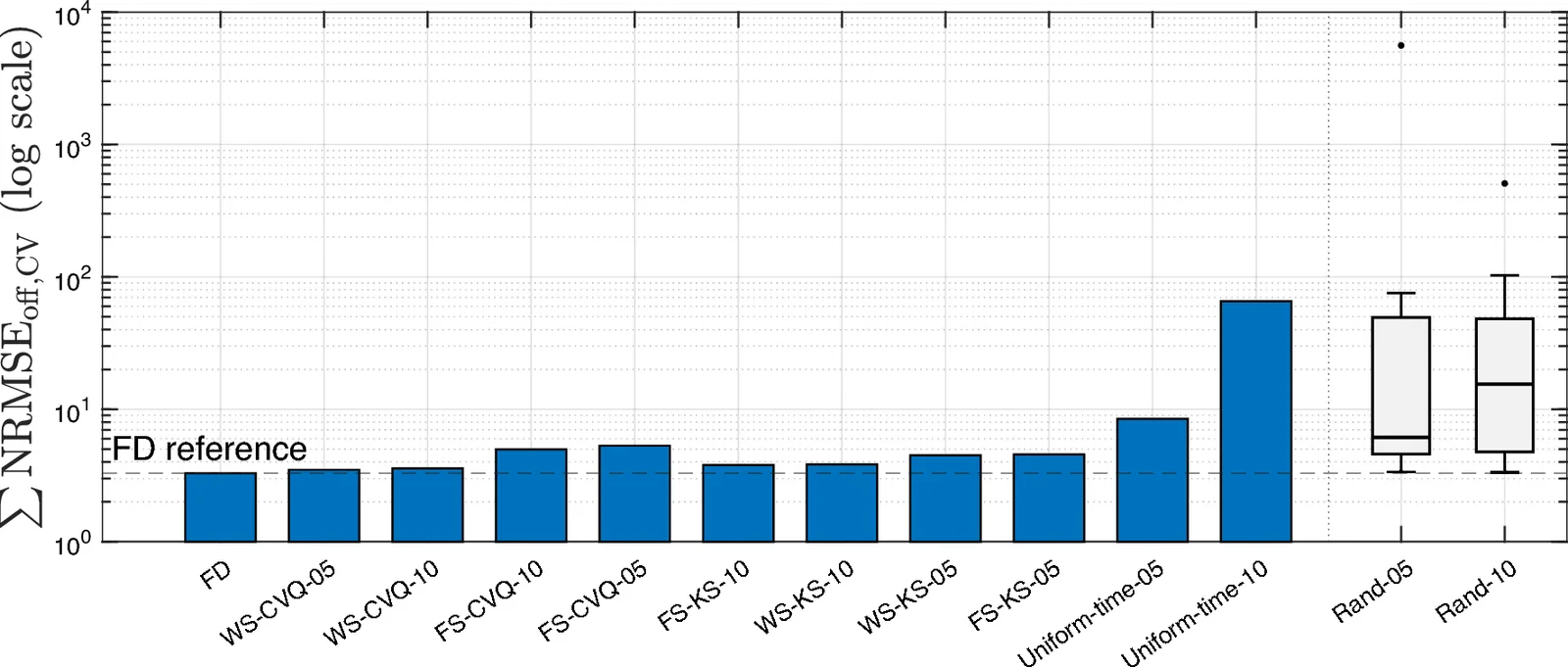
Cross-validation (CV) benchmark of reduced sampling strategies based on the summed NRMSE of the four offline analytes on the independent F2 experiment. Deterministic strategies are shown as single values, whereas RS is shown as boxplots over 20 stochastic realizations. The logarithmic y-axis visualizes both competitive random subsets and severe failure cases. The full-data reference (FD) is shown as dashed reference line

Figure 5 depicts the CV evaluation using $\sum \textrm{NRMSE}_{\textrm{off,CV}}$. Detailed analyte-wise values for the original FD and CVQ scenarios are presented in Table 8. The KS and UI baselines are reported separately in Table 9, whereas the RS baselines are summarized in Table 10. FD remains the best full-data reference for the offline analytes ($\sum \textrm{NRMSE}_{\textrm{off,CV}}=3.29$). WS-CVQ-05 and WS-CVQ-10 remain closest to this reference among the CVQ-based reduced strategies, with values of 3.49 and 3.58. The KS baselines further confirm that spectral diversity is informative for RGSS, with FS-KS-10 and WS-KS-10 yielding competitive CV errors of 3.79 and 3.83, respectively. In contrast, non-spectral UI sampling is less robust. UI-05 yields 8.47, whereas UI-10 fails to generalize to F2 and reaches 65.57, mainly due to glucose and ammonium.

The summed offline NRMSE provides a compact multi-analyte ranking, but it does not imply that one reduced strategy is optimal for every analyte. For example, WS-CVQ-05 achieved the lowest CDW error among all scenarios ($\textrm{NRMSE}_{\textrm{CV}}=0.13$), whereas WS-CVQ-10 achieved the lowest glucose error (0.77). FD and WS-KS-05 jointly achieved the lowest ammonium error (0.68), and FS-KS-10 achieved the lowest phosphate error (0.85). Thus, the preferred subset-selection strategy can differ between analytes, reflecting differences in concentration range, spectral prominence, measurement uncertainty, and process excitation.

RS occasionally generates competitive subsets, but its performance is highly variable. For RS-05, the median $\sum \textrm{NRMSE}_{\textrm{off,CV}}$ is 6.14 with an IQR of 4.84–47.76 and a severe outlier exceeding $10^3$. For RS-10, the corresponding median is 15.48 with an IQR of 5.07–44.34 and a maximum exceeding $10^2$. Thus, RS can succeed by chance but is not reliable for small subset sizes.

**Table 8:** NRMSE-CV results for four offline analytes and two online measurements, dissolved oxygen tension (DOT) and cumulative base volume ($m_\textrm{B}$) under five sampling strategies

Analyte	FD	FS-CVQ-10	FS-CVQ-05	WS-CVQ-10	WS-CVQ-05
*Offline analytes (NRMSE-CV)*					
$c_\textrm{CDW}$	0.43	0.26	1.14	0.33	0.13
$c_\textrm{Glc}$	0.93	1.58	1.05	0.77	0.94
$c_\textrm{Am}$	0.68	1.87	1.86	1.24	1.16
$c_\textrm{Ph}$	1.25	1.27	1.26	1.24	1.26
$\sum (\mathrm {NRMSE_{off}})$	**3**.**29**	**4**.**98**	**5**.**31**	**3**.**58**	**3**.**49**
*Online measurements (NRMSE-CV)*					
*DOT*	2.71	3.20	2.83	3.81	3.92
$m_\textrm{B}$	0.36	0.35	1.08	0.60	0.59
$\sum (\mathrm {NRMSE_{all}})$	**6**.**37**	**8**.**54**	**9**.**22**	**7**.**99**	**8**.**00**

Shown in bold are the summed offline NRMSE values $\sum (\mathrm {NRMSE_{off}})$, and the total $\sum (\mathrm {NRMSE_{all}})$

**Table 9:** NRMSE-CV benchmark for KS and uniform-in-time (UI) baseline strategies on the independent F2 experiment

Analyte	FS-KS-10	FS-KS-05	WS-KS-10	WS-KS-05	UI-10	UI-05
*Offline analytes (NRMSE-CV)*						
$c_\textrm{CDW}$	0.64	0.27	1.16	1.83	2.52	1.66
$c_\textrm{Glc}$	1.06	1.20	1.07	1.08	33.25	2.30
$c_\textrm{Am}$	1.24	1.11	0.71	0.68	25.74	1.34
$c_\textrm{Ph}$	0.85	1.98	0.89	0.92	4.06	3.18
$\sum (\mathrm {NRMSE_{off}})$	**3**.**79**	**4**.**56**	**3**.**83**	**4**.**49**	**65**.**57**	**8**.**47**
*Online measurements (NRMSE-CV)*						
*DOT*	1.48	1.47	1.46	1.47	1.76	1.45
$m_\textrm{B}$	0.38	1.74	0.09	0.01	0.02	0.07
$\sum (\mathrm {NRMSE_{all}})$	**5**.**65**	**7**.**77**	**5**.**39**	**5**.**97**	**67**.**35**	**10**.**00**

Shown in bold are the summed offline NRMSE values $\sum (\mathrm {NRMSE_{off}})$, and the total $\sum (\mathrm {NRMSE_{all}})$

**Table 10:** Random NRMSE-CV benchmark for RS-10 and RS-05 on the independent F2 experiment. Values are reported as median and interquartile range (IQR) over 20 random runs

Analyte	RS-10	IQR	RS-05	IQR
*Offline analytes (NRMSE-CV)*				
$c_\textrm{CDW}$	3.88	[1.34–7.70]	3.14	[1.35–6.11]
$c_\textrm{Glc}$	5.02	[1.01–12.79]	1.15	[1.03–8.72]
$c_\textrm{Am}$	1.28	[1.19–5.29]	1.28	[0.79–6.14]
$c_\textrm{Ph}$	1.61	[1.01–9.56]	1.14	[0.91–2.80]
$\sum (\mathrm {NRMSE_{off}})$	**15**.**48**	**[5.07–44.34]**	**6**.**14**	**[4.84–47.76]**
*Online measurements (NRMSE-CV)*				
*DOT*	1.48	[1.48–1.49]	1.48	[1.46–1.48]
$m_\textrm{B}$	0.07	[0.02–0.22]	0.09	[0.03–0.34]
$\sum (\mathrm {NRMSE_{all}})$	**17**.**27**	**[6.69–62.29]**	**9**.**62**	**[6.75–49.25]**

Shown in bold are the summed offline NRMSE values $\sum (\mathrm {NRMSE_{off}})$, and the total $\sum (\mathrm {NRMSE_{all}})$

For the CVQ scenarios, (DOT) NRMSE values ranged from 2.71 to 3.92 and ($m_\textrm{B}$) errors from 0.35 to 1.08. WS-KS-10 and FS-KS-10 achieved lowest all-output CV errors of 5.39 and 5.65, respectively, partly because the online variables were reproduced well. The full-data reference retained ($\sum \textrm{NRMSE}_{\textrm{all,CV}}$ = 6.37) among these scenarios, followed by WS-CVQ-10 (7.99), WS-CVQ-05 (8.00), FS-CVQ-10 (8.54), and FS-CVQ-05 (9.22). In contrast, UI-10 remained poor also in ($\sum \textrm{NRMSE}_{\textrm{all,CV}}$) (67.35) due to the large offline glucose and ammonium errors. RS-05 and RS-10 yielded median all-output CV errors of 9.62 [6.75–49.25] and 17.27 [6.69–62.29], respectively. Since the subset-selection strategies reduce only the offline reference assays, these all-output values are interpreted as secondary consistency metrics rather than as the primary ranking criterion.

### Practical identifiability analysis

The practical identifiability analysis was performed for the main CVQ scenarios and the FD reference, because these constitute the primary RGSS contribution and were evaluated in detail by SbS. An overview of the practical identifiability of each parameter after PI is provided in Table 11, and the corresponding parameter estimates are listed in Table 12. Overall, $\mu _{\text {Maint,max}}$, $Y_{\text {Am/CDW}}$ and $Y_{\text {B/Am}}$ are practically non-identifiable in these scenarios, whereas $Y_{\text {O/Glc}}$ is additionally non-identifiable in the FS-CVQ-10 and FS-CVQ-05 scenarios. This indicates that some maintenance- and yield-related parameters remain only weakly constrained by the present dataset, even when all available offline data are used.

**Table 11:**
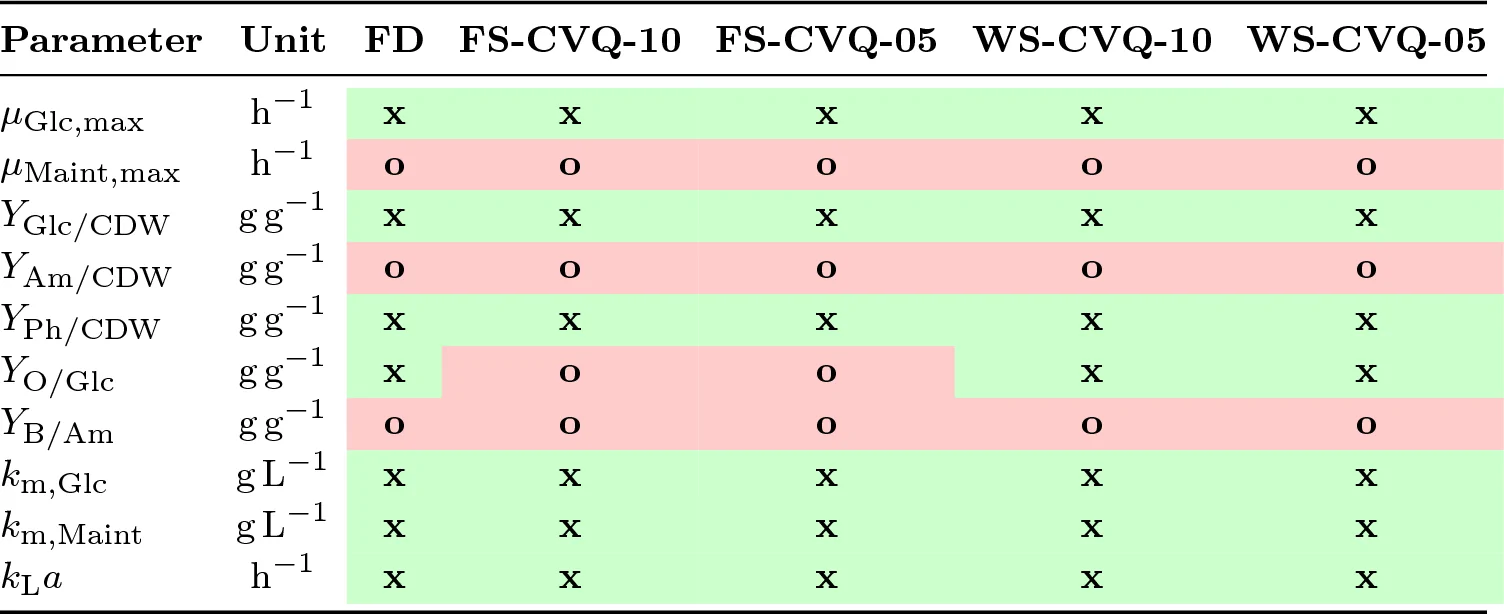
Practical identifiability analysis based on sensitivity-based parameter subset selection (x: identifiable, o: not identifiable)

**Table 12:** Model parameter estimates $\hat{\boldsymbol{\theta }}$ obtained by PI on F1 using FD, FS-CVQ-10, FS-CVQ-05, WS-CVQ-10, and WS-CVQ-05, rounded to significant figures

Parameter	Unit	FD	FS-CVQ-10	FS-CVQ-05	WS-CVQ-10	WS-CVQ-05
$\mu _{\textrm{Glc,max}}$	h$^{-1}$	0.42	0.35	0.31	0.35	0.33
$\mu _{\textrm{Maint,max}}$	h$^{-1}$	0.19	0.02	0.07	0.12	0.06
$Y_{\mathrm {Glc/CDW}}$	g g$^{-1}$	10.78	11.53	11.06	10.87	12.77
$Y_{\mathrm {Am/CDW}}$	g g$^{-1}$	0.09	0.11	0.11	0.10	0.14
$Y_{\mathrm {Ph/CDW}}$	g g$^{-1}$	0.04	0.04	0.03	0.03	0.05
$Y_{\mathrm {O/Glc}}$	g g$^{-1}$	0.10	0.13	0.12	0.12	0.16
$Y_{\mathrm {B/Am}}$	g g$^{-1}$	0.05	0.04	0.04	0.04	0.03
$k_{\textrm{m,Glc}}$	g L$^{-1}$	8.34	4.59	5.32	5.44	1.32
$k_{\textrm{m,Maint}}$	g L$^{-1}$	11.00	2.02	2.34	2.06	2.61
$k_{\textrm{L}}a$	h$^{-1}$	532	405	524	513	571

## Discussion

The presented results support RGSS as a spectrally informed framework for prioritizing offline reference assays before nonlinear model calibration. The central question was whether inline Raman spectra provide useful information for deciding which stored candidate samples should undergo costly offline analytics. The baseline comparisons with UI and RS strengthen this interpretation. CVQ-based RGSS remains competitive with the full-data reference, KS confirms that spectral diversity alone is already informative, and the UI and RS baselines show that simple data reduction without spectral information is less reliable.

The poor CV performance of UI-10 can be explained by the irregular availability of offline candidate samples. Because no offline samples were available during the overnight gap between approximately 8 and 22 h, several equally spaced target times were projected onto neighbouring available samples at the edges of this gap, resulting in clustered selections such as 22–23 h and 34–35 h (Fig. 3). Thus, uniform-in-time sampling did not necessarily provide a uniformly informative subset with respect to process dynamics. This effect was particularly critical for glucose and ammonium, where transitions between depletion, feeding, and concentration changes strongly affect PI and CV performance.

RS further illustrates that some random subsets performed well, indicating that informative sample combinations can occur by chance. However, the large spread and severe failure cases show that random selection is not a robust strategy for resource-limited calibration with irregularly spaced candidate set. Increasing the number of randomly selected samples from five to ten did not result in a consistent improvement. The RS-05 and RS-10 realizations were generated independently and were not nested. Consequently, an RS-10 realization did not necessarily contain the favourable samples of a corresponding RS-05 realization. Additional randomly selected samples could still be distributed redundantly or unfavourably across process phases and could introduce conflicting information under model mismatch and nonlinear PI. This supports the central motivation of RGSS, that inline Raman spectra provide a process-informed, model-free criterion for prioritizing stored candidate samples before offline reference analytics. Nevertheless, this result should not be interpreted as a general disadvantage of using more calibration samples, but as evidence that subset size alone does not guarantee an informative calibration set.

The difference between FS- and WS-based RGSS is central for interpreting the results. In FS-CVQ and FS-KS, the same Raman fingerprint region at each time point is used to select one common set of samples for all four analytes. As a consequence, the selected sampling times can be dominated by the strongest global spectral changes, for example biomass increase, glucose dynamics, scattering, or turbidity-related effects. Furthermore, FS-based methods are less flexible because one common subset must serve all four analytes simultaneously. In contrast, WS-CVQ and WS-KS use analyte-specific Raman bands to select samples independently for each output. Thus, the same nominal number of reference assays per analyte can provide a broader temporal distribution across the multi-output PI problem. This analyte-specific temporal flexibility is a key advantage of WS-based RGSS and helps to explain why WS-CVQ-05 remains close to FD in NRMSE-CV despite the strong assay reduction.

The analytes also differ substantially in concentration range and spectral dominance. Glucose is present at higher concentration and can contribute strongly to spectral variance, whereas ammonium and phosphate are present at lower concentrations and have fewer or weaker isolated Raman bands. This helps explain why FS RGSS may be dominated by glucose, biomass, or scattering-related changes and does not necessarily select the most informative samples for each analyte. It is noteworthy that the CV results for $c_\textrm{Ph}$ remain comparatively challenging across several methods. This may be related to uncertainties in the initial phosphate concentration for F2, which could be lower than assumed in the model. Such mismatches in initial conditions can limit the achievable prediction accuracy for this analyte in CV. Accordingly, $\sum \textrm{NRMSE}_{\textrm{off}}$ is interpreted as an overall multi-analyte assessment criterion for the complete PI problem, rather than as evidence of a universally optimal sampling strategy for each individual analyte.

The nonlinear PI problem involves stochastic optimization components. To keep the comparison between subset-selection strategies attributable to the selected samples rather than to optimizer variability, identical PI settings, fixed random seeds, and the same precomputed initialization strategy were used for all deterministic scenarios. RS baseline was repeated over 20 random subset-selection runs to quantify the variability introduced by stochastic sample selection itself. A full optimizer-seed uncertainty analysis for every subset-selection scenario is computationally expensive and remains an important extension for future work.

The present implementation evaluates RGSS for nonlinear PI of a mechanistic *S. cerevisiae* model. The underlying principle is more general and could also be used with hybrid or data-driven models that map measurement data and process variables. A detailed quantitative comparison between RGSS-based mechanistic soft sensors and purely data-driven soft-sensing architectures should be addressed in future studies.

## Conclusion and outlook

This study introduced Raman-guided sample subset selection (RGSS) as a model-free strategy for prioritizing informative offline reference assays before nonlinear PI. CVQ and KS retained offline CV accuracies close to the full-data FD reference while using only five selected reference assays per analyte. In contrast, RS and UI subsets were less reliable because they do not use spectral information and can fail under irregular sampling schedules. FS-based RGSS methods are not generally unsuitable, but they are less flexible because a single common subset must serve all analytes. WS-based RGSS methods can distribute the same number of reference assays per analyte across different process phases, thereby providing additional temporal information for multi-output PI.

RGSS is particularly useful in early development stages, where reliable model-based experimental design is not yet feasible because model parameters and even model structure may be uncertain. While mbDoE optimizes experiments based on an assumed process model and may therefore degrade under PMM and parameter uncertainty, RGSS performs subset selection directly on measured spectral time series and is largely independent of the mechanistic model at the selection stage. For automated sampling and at-line analyser platforms that can generate dense sample streams over extended cultivations, RGSS provides a practical decision layer for selecting which stored candidate samples should undergo costly reference analytics.

Future work should combine RGSS with mbDoE to improve the practical identifiability of parameters that remained weakly constrained in this study, in particular maintenance- and yield-related parameters. Further extensions include systematic preprocessing and wavelength-selection studies, incorporation of domain expertise on Raman band assignments, application to additional organisms and process conditions, and integration with Raman-based soft sensing or online state estimation. Such developments would connect spectrally guided offline assay prioritization with model-based state estimation, control, and optimization.

## Supplementary Information

Below is the link to the electronic supplementary material.

Supplementary file 1 (pdf 8404 KB)

## Data Availability

No datasets were generated or analysed during the current study.

## Code availability

Compact reference implementations of the CVQ- and KS-based sample subset-selection procedures are provided in Online Resource 1.

## References

[1] Vassiliadis VS, Mappas V, Espaas TA, Dorneanu B, Isafiade A, Möller K, Arellano-Garcia H (2024) Reloading process systems engineering within chemical engineering. Chem Eng Res Des. 10.1016/j.cherd.2024.07.066

[2] Nielsen J, Tillegreen CB, Petranovic D (2022) Innovation trends in industrial biotechnology. Trends Biotechnol 40(10):1160–1172. 10.1016/j.tibtech.2022.03.007 35459568

[3] Scott A (2022) Carving out a giant. C&EN Glob Enterp 100(13):23–24. 10.1021/cen-10013-feature5

[4] Mears L, Stocks SM, Sin G, Gernaey KV (2017) A review of control strategies for manipulating the feed rate in fed-batch fermentation processes. J Biotechnol 245:34–46. 10.1016/j.jbiotec.2017.01.008 28179156

[5] Abt V, Barz T, Cruz Bournazou MN, Herwig C, Kroll P, Möller J, Pörtner R, Schenkendorf R (2018) Model-based tools for optimal experiments in bioprocess engineering. Curr Opin Chem Eng 22:244–252. 10.1016/j.coche.2018.11.007

[6] Mears L, Stocks SM, Albaek MO, Sin G, Gernaey KV (2017) Mechanistic fermentation models for process design, monitoring, and control. Trends Biotechnol 35(10):914–924. 10.1016/j.tibtech.2017.07.002 28838636

[7] Kroll P, Hofer A, Ulonska S, Kager J, Herwig C (2017) Model-based methods in the biopharmaceutical process lifecycle. Pharm Res 34(12):2596–2613. 10.1007/s11095-017-2308-y 29168076 PMC5736780

[8] Krämer D, Wilms T, King R (2020) Model-based process optimization for the production of macrolactin D by *Paenibacillus polymyxa*. Processes 8(7):752. 10.3390/pr8070752

[9] King R (2021) Control of biotechnological processes. Encycl Syst Control 11:298–305. 10.1007/978-3-030-44184-5_252

[10] Bayer B, Duerkop M, Pörtner R, Möller J (2023) Comparison of mechanistic and hybrid modeling approaches for characterization of a cho cultivation process: Requirements, pitfalls and solution paths. Biotechnol J 18(1):2200381. 10.1002/biot.202200381 36382343

[11] Rathore AS, Sarin D (2024) What should next-generation analytical platforms for biopharmaceutical production look like? Trends Biotechnol 42(3):282–292. 10.1016/j.tibtech.2023.08.008 37775418

[12] Etit D, Meramo S, Ögmundarson Ó, Jensen MK, Sukumara S (2024) Can biotechnology lead the way toward a sustainable pharmaceutical industry? Curr Opin Biotechnol 87:103100. 10.1016/j.copbio.2024.103100 38471403

[13] Cruz Bournazou MN, Barz T, Nickel D, Lopez Cárdenas DC, Glauche F, Knepper A, Neubauer P (2017) Online optimal experimental re-design in robotic parallel fed-batch cultivation facilities. Biotechnol Bioeng 114(3):610–619. 10.1002/bit.26192 27696353

[14] Nickel DB, Cruz Bournazou MN, Wilms T, Neubauer P, Knepper A (2017) Online bioprocess data generation, analysis, and optimization for parallel fed-batch fermentations in milliliter scale. Technical report, Wiley Online Library. 10.1002/elsc.201600035 PMC6999281 32624747

[15] Hemmerich J, Noack S, Wiechert W, Oldiges M (2018) Microbioreactor systems for accelerated bioprocess development. Biotechnol J 13(4):1700141. 10.1002/biot.201700141 29283217

[16] Walter E, Pronzato L (1997) Identification of parametric models: from experimental data. Communications and control engineering. Springer

[17] Franceschini G, Macchietto S (2008) Model-based design of experiments for parameter precision: state of the art. Chem Eng Sci 63(19):4846–4872. 10.1016/j.ces.2007.11.034

[18] Paquet-Durand O, Zettel V, Hitzmann B (2016) A bootstrap-based method for optimal design of experiments. J Chemom 30(10):567–574. 10.1002/cem.2820

[19] Heine T, Kawohl M, King R (2008) Derivative-free optimal experimental design. Chem Eng Sci 63(19):4873–4880. 10.1016/j.ces.2008.01.030

[20] Daume S, Kager J, Herwig C (2019) Time resolved sensitivity & identifiability analysis for directed parametrization of highly dynamic models. In: Computer aided chemical engineering vol. 46, pp. 1111–1116. Elsevier, Amsterdam. 10.1016/B978-0-12-818634-3.50186-7

[21] Anane E, López C, DC, Barz T, Sin G, Gernaey KV, Neubauer P, Cruz Bournazou, MN, (2019) Output uncertainty of dynamic growth models: effect of uncertain parameter estimates on model reliability. Biochem Eng J 150:107247. 10.1016/j.bej.2019.107247

[22] Buckley K, Ryder AG (2017) Applications of Raman spectroscopy in biopharmaceutical manufacturing: a short review. Appl Spectrosc 71(6):1085–1111. 10.1177/0003702817703270 28534676

[23] Schalk R, Heintz A, Braun F, Iacono G, Rädle M, Gretz N, Methner F-J, Beuermann T (2019) Comparison of Raman and mid-infrared spectroscopy for real-time monitoring of yeast fermentations: a proof-of-concept for multi-channel photometric sensors. Appl Sci 9(12):2472. 10.3390/app9122472

[24] Rolinger L, Ruedt M, Hubbuch J (2020) A critical review of recent trends, and a future perspective of optical spectroscopy as PAT in biopharmaceutical downstream processing. Anal Bioanal Chem 412:2047–2064. 10.1007/s00216-020-02407-z 32146498 PMC7072065

[25] Esmonde-White KA, Cuellar M, Lewis IR (2022) The role of Raman spectroscopy in biopharmaceuticals from development to manufacturing. Anal Bioanal Chem 414(2):969–991. 10.1007/s00216-021-03727-4 34668998 PMC8724084

[26] Bocklitz T, Walter A, Hartmann K, Rösch P, Popp J (2011) How to pre-process Raman spectra for reliable and stable models? Anal Chim Acta 704(1–2):47–56. 10.1016/j.aca.2011.06.043 21907020

[27] Guo S, Bocklitz T, Popp J (2016) Optimization of Raman-spectrum baseline correction in biological application. Analyst 141(8):2396–2404. 10.1039/C6AN00041J 26907832

[28] Guo S, Popp J, Bocklitz T (2021) Chemometric analysis in Raman spectroscopy from experimental design to machine learning-based modeling. Nat Protoc 16(12):5426–5459. 10.1038/s41596-021-00620-3 34741152

[29] Esmonde-White KA, Cuellar M, Uerpmann C, Lenain B, Lewis IR (2017) Raman spectroscopy as a process analytical technology for pharmaceutical manufacturing and bioprocessing. Anal Bioanal Chem 409(3):637–649. 10.1007/s00216-016-9824-1 27491299 PMC5233728

[30] Claßen J, Aupert F, Reardon KF, Solle D, Scheper T (2017) Spectroscopic sensors for in-line bioprocess monitoring in research and pharmaceutical industrial application. Anal Bioanal Chem 409:651–666. 10.1007/s00216-016-0068-x 27900421

[31] Abu-Absi NR, Martel RP, Lanza AM, Clements SJ, Borys MC, Li ZJ (2014) Application of spectroscopic methods for monitoring of bioprocesses and the implications for the manufacture of biologics. Pharm Bioprocess 2(3):267–284. 10.4155/PBP.14.24

[32] Kern S, Meyer K, Guhl S, Gräßer P, Paul A, King R, Maiwald M (2018) Online low-field NMR spectroscopy for process control of an industrial lithiation reaction–automated data analysis. Anal Bioanal Chem 410:3349–3360. 10.1007/s00216-018-1020-z 29616294

[33] Shaw AD, Kaderbhai N, Jones A, Woodward AM, Goodacre R, Rowland JJ, Kell DB (1999) Noninvasive, on-line monitoring of the biotransformation by yeast of glucose to ethanol using dispersive Raman spectroscopy and chemometrics. Appl Spectrosc 53(11):1419–1428. 10.1366/0003702991945777

[34] Kögler M, Paul A, Anane E, Birkholz M, Bunker A, Viitala T, Maiwald M, Junne S, Neubauer P (2018) Comparison of time-gated surface-enhanced Raman spectroscopy (TG-SERS) and classical SERS based monitoring of Escherichia coli cultivation samples. Biotechnol Prog 34(6):1533–1542. 10.1002/btpr.2665 29882305

[35] Yang N, Guerin C, Kokanyan N, Perré P (2024) Raman spectroscopy applied to online monitoring of a bioreactor: tackling the limit of detection. Spectrochim Acta Part A Mol Biomol Spectrosc 304:123343. 10.1016/j.saa.2023.123343 37690399

[36] Bauckhage C (2020) Numpy/scipy recipes for data science: Mean discrepancy minimization for vector quantization. researchgate. net, Feb. Accessed 1 Jul 2023

[37] Bauckhage C (2020) Numpy/scipy recipes for data science: subset-constrained vector quantization via mean discrepancy minimization. researchgate. net, June, 1–4. Accessed 1 Jul 2023

[38] Krause J, Günder M, Schulz D, Gruna R (2021) New active learning algorithms for near-infrared spectroscopy in agricultural applications. at-Automatisierungstechnik 69(4):297–306. 10.1515/auto-2020-0143

[39] Bian X, Yang W, Zhang K, Zhang Q, Tian W, Kollenburg G (2025) A review on sample subset selection methods for multivariate modelling. Chemom Intell Lab Syst. 10.1016/j.chemolab.2025.105493

[40] Kennard RW, Stone LA (1969) Computer aided design of experiments. Technometrics 11(1):137–148. 10.1080/00401706.1969.10490666

[41] Galvao RKH, Araujo MCU, José GE, Pontes MJC, Silva EC, Saldanha TCB (2005) A method for calibration and validation subset partitioning. Talanta 67(4):736–740. 10.1016/j.talanta.2005.03.025 18970233

[42] Pétillot L, Pewny F, Wolf M, Sanchez C, Thomas F, Sarrazin J, Fauland K, Katinger H, Javalet C, Bonneville C (2020) Calibration transfer for bioprocess Raman monitoring using Kennard stone piecewise direct standardization and multivariate algorithms. Eng Rep 2(11):12230. 10.1002/eng2.12230

[43] Guisasola A, Baeza JA, Carrera J, Sin G, Vanrolleghem PA, Lafuente J (2006) The influence of experimental data quality and quantity on parameter estimation accuracy: Andrews inhibition model as a case study. Educ Chem Eng 1(1):139–145. 10.1205/ece06016

[44] Villaverde AF, Fröhlich F, Weindl D, Hasenauer J, Banga JR (2019) Benchmarking optimization methods for parameter estimation in large kinetic models. Bioinformatics 35(5):830–838. 10.1093/bioinformatics/bty736 30816929 PMC6394396

[45] Wilms T, Knorn S (2026) Identifiability-guided Bound-tightening metaheuristics for parameter identification under plant–model mismatch. In: Submitted to 12th International Conference on Control, Decision and Information Technologies (CoDIT 2026). Accepted

[46] Stein M (1987) Large sample properties of simulations using Latin hypercube sampling. Technometrics 29(2):143–151. 10.1080/00401706.1987.10488205

[47] McKay MD, Beckman RJ, Conover WJ (2000) A comparison of three methods for selecting values of input variables in the analysis of output from a computer code. Technometrics 42(1):55–61. 10.1080/00401706.2000.10485979

[48] López C, DC, Barz T, Körkel S, Wozny G (2015) Nonlinear ill-posed problem analysis in model-based parameter estimation and experimental design. Comput Chem Eng 77:24–42. 10.1016/j.compchemeng.2015.03.002

[49] Barz T, Sommer A, Wilms T, Neubauer P, Cruz Bournazou MN (2018) Adaptive optimal operation of a parallel robotic liquid handling station. IFAC-PapersOnLine 51(2):765–770. 10.1016/j.ifacol.2018.04.006

[50] Nevoigt E (2008) Progress in metabolic engineering of *Saccharomyces cerevisiae*. Microbiol Mol Biol Rev 72(3):379–412. 10.1128/mmbr.00025-07 18772282 PMC2546860

[51] Lian J, Mishra S, Zhao H (2018) Recent advances in metabolic engineering of *Saccharomyces cerevisiae*: new tools and their applications. Metab Eng 50:85–108. 10.1016/j.ymben.2018.04.011 29702275

[52] Herold S, Krämer D, Violet N, King R (2017) Rapid process synthesis supported by a unified modular software framework. Eng Life Sci 17(11):1202–1214. 10.1002/elsc.201600020 32624748 PMC6999271

[53] Krämer D, King R (2019) A hybrid approach for bioprocess state estimation using NIR spectroscopy and a sigma-point Kalman filter. J Process Control 82:91–104. 10.1016/j.jprocont.2017.11.008

[54] Zhang Z-M, Chen S, Liang Y-Z (2010) Baseline correction using adaptive iteratively reweighted penalized least squares. Analyst 135(5):1138–1146. 10.1039/B922045C 20419267

[55] Morais CL, Santos MC, Lima KM, Martin FL (2019) Improving data splitting for classification applications in spectrochemical analyses employing a random-mutation kennard-stone algorithm approach. Bioinformatics 35(24):5257–5263. 10.1093/bioinformatics/btz421 31116391 PMC6954661

[56] Dwivedi R, Mackey L (2024) Kernel thinning. J Mach Learn Res 25(152):1–77. 10.48550/arXiv.2105.0584241334350

[57] Gretton A, Borgwardt KM, Rasch MJ, Schölkopf B, Smola A (2012) A kernel two-sample test. J Mach Learn Res 13(25):723–773 (http://hdl.handle.net/20.500.11850/87955)

[58] Schölkopf B (2001) The kernel trick for distances. In: Leen T, Dietterich T, Tresp V (eds) Advances in neural information processing systems, vol 13. MIT Press, pp 301–307

[59] Scott DW (2012) Multivariate density estimation and visualization. Springer, pp 549–569. 10.1007/978-3-642-21551-3_19

[60] Krämer D, King R (2016) On-line monitoring of substrates and biomass using near-infrared spectroscopy and model-based state estimation for enzyme production by *S. cerevisiae*. IFAC-PapersOnLine 49(7):609–614. 10.1016/j.ifacol.2016.07.235

[61] Enfors S-O, Hedenberg J, Olsson K (1990) Simulation of the dynamics in the baker’s yeast process. Bioprocess Eng 5:191–198. 10.1007/BF00376225

[62] Pham HTB, Larsson G, Enfors S-O (1998) Growth and energy metabolism in aerobic fed-batch cultures of *Saccharomyces cerevisiae*: simulation and model verification. Biotechnol Bioeng 60(4):474–482. 10.1002/(sici)1097-0290(19981120)60:4<474::aid-bit9>3.0.co;2-j 10099453

[63] Heine T, Kawohl M, King R (2003) Parameterschätzung für nichtlineare dynamische modelle auf basis stark verrauschter, statistisch unvollständig bestimmter messdaten. Chem Ing Tec 75(5):543–550. 10.1002/cite.200390105

[64] Raue A, Kreutz C, Maiwald T, Bachmann J, Schilling M, Klingmüller U, Timmer J (2009) Structural and practical identifiability analysis of partially observed dynamical models by exploiting the profile likelihood. Bioinformatics 25(15):1923–1929. 10.1093/bioinformatics/btp358 19505944

[65] Díaz-Seoane S, Rey Barreiro X, Villaverde AF (2023) Strike-goldd 4.0: user-friendly, efficient analysis of structural identifiability and observability. Bioinformatics 39(1):748. 10.1093/bioinformatics/btac748 PMC9805590 36398887

[66] Villaverde AF, Barreiro A, Papachristodoulou A (2016) Structural identifiability of dynamic systems biology models. PLoS Comput Biol 12(10):1005153. 10.1371/journal.pcbi.1005153 PMC5085250 27792726

[67] Villaverde AF, Tsiantis N, Banga JR (2019) Full observability and estimation of unknown inputs, states and parameters of nonlinear biological models. J R Soc Interface 16(156):20190043. 10.1098/rsif.2019.0043 31266417 PMC6685009

[68] Virtanen T, Reinikainen S-P, Lahti J, Mänttäri M, Kallioinen M (2018) Visual tool for real-time monitoring of membrane fouling via Raman spectroscopy and process model based on principal component analysis. Sci Rep 8(1):11057. 10.1038/s41598-018-29268-y PMC6056556 30038320

[69] Rossberg N, Gautam R, Komolibus K, O’Sullivan B, Visentin A (2025) Explainable ai-based feature selection approaches for Raman spectroscopy. Diagnostics 15(16):2063. 10.3390/diagnostics15162063 40870915 PMC12385318

[70] De Iseppi A, Lomolino G, Marangon M, Curioni A (2020) Current and future strategies for wine yeast lees valorization. Food Res Int 137:109352. 10.1016/j.foodres.2020.109352 33233056

[71] Lemma T, Wang J, Arstila K, Hytönen VP, Toppari JJ (2019) Identifying yeasts using surface enhanced Raman spectroscopy. Spectrochim Acta Part A Mol Biomol Spectrosc 218:299–307. 10.1016/j.saa.2019.04.010 31005737

[72] Lipke PN, Ovalle R (1998) Cell wall architecture in yeast: new structure and new challenges. J Bacteriol 180(15):3735–3740. 10.1128/jb.180.15.3735-3740.1998 9683465 PMC107352

[73] Latgé J-P (2007) The cell wall: a carbohydrate armour for the fungal cell. Mol Microbiol 66(2):279–290. 10.1111/j.1365-2958.2007.05872.x 17854405

[74] Ball A (1993) Carbohydrates and sugars. In: Biochemistry Labfax, pp. 305–315. Elsevier, Amsterdam. 10.1016/B978-0-12-167340-6.50020-3

[75] Wan M, Wang M, Zhao Y, Deng H, Tan C, Lin S, Kong Y, Tong Y, Meng X (2021) Extraction of mannoprotein from *Saccharomyces cerevisiae* and analysis of its chemical composition and molecular structure. Int J Biol Macromol 193:2252–2259. 10.1016/j.ijbiomac.2021.11.057 34785200

[76] Iversen JA, Berg RW, Ahring BK (2014) Quantitative monitoring of yeast fermentation using Raman spectroscopy. Anal Bioanal Chem 406:4911–4919. 10.1007/s00216-014-7897-2 24996999

[77] Pontius K, Praticò G, Larsen FH, Skov T, Arneborg N, Lantz AE, Bevilacqua M (2020) Fast measurement of phosphates and ammonium in fermentation-like media: a feasibility study. New Biotechnol 56:54–62. 10.1016/j.nbt.2019.11.006 31770609

[78] Noothalapati H, Sasaki T, Kaino T, Kawamukai M, Ando M, Hamaguchi H-O, Yamamoto T (2016) Label-free chemical imaging of fungal spore walls by Raman microscopy and multivariate curve resolution analysis. Sci Rep 6(1):27789. 10.1038/srep27789 27278218 PMC4899791

[79] Freire PTC, Barboza FM, Lima JA, Melo FEA, Filho JM (2017) Raman spectroscopy of amino acid crystals. In: Raman spectroscopy and applications. IntechOpen, London. Chap. 10. 10.5772/65480

[80] Rösch P, Harz M, Schmitt M, Popp J (2005) Raman spectroscopic identification of single yeast cells. J Raman Spectrosc 36(5):377–379. 10.1002/jrs.1312

