# Supplementary material for: Raman-guided sample subset selection for cost-efficient offline calibration in bioprocesses
Source: Bioprocess Biosyst Eng. 2026 Aug 12;49(10):2651–71. doi: 10.1007/s00449-026-03402-x (PMC13582237; doi:10.1007/s00449-026-03402-x)
Supplement: Supplementary file 1 [file 449_2026_3402_MOESM1_ESM.pdf]

Bioprocess and Biosystems Engineering

Supplementary material

Raman-guided Sample Subset Selection for Cost-efficient Offline Calibration in Bioprocesses

Terrance Wilms[a], Fabian Schwenke[a], Rudibert King[a], Steffi Knorn[a]

[a] Technische Universität Berlin, Chair of Control, Hardenbergstr. 36a, 10623 Berlin, Germany

Contents

1 Supplementary Figures **2**
1.1 Raman spectral overview 2
1.2 Parameter Identification 6

2 Supplementary Tables **11**

3 Reference MATLAB implementations **16**

Supplementary Material

This document contains supplementary figures, tables, and reference MATLAB implementations referenced in the main manuscript as **Figures S1–S13**, **Tables S1–S6**, and **Listings 1–2**.

1 Supplementary Figures

1.1 Raman spectral overview

Figures S1–S4 complement the representative Raman spectra shown in Fig. 2 of the main manuscript. For each candidate offline sampling time, 110 consecutive airPLS-corrected in-line Raman acquisitions were extracted over an approximately 10-min interval and aggregated into one pointwise mean spectrum. Figure S1 shows the resulting 22 mean candidate spectra together with the literature-derived wavenumber regions used for the WS feature representations.

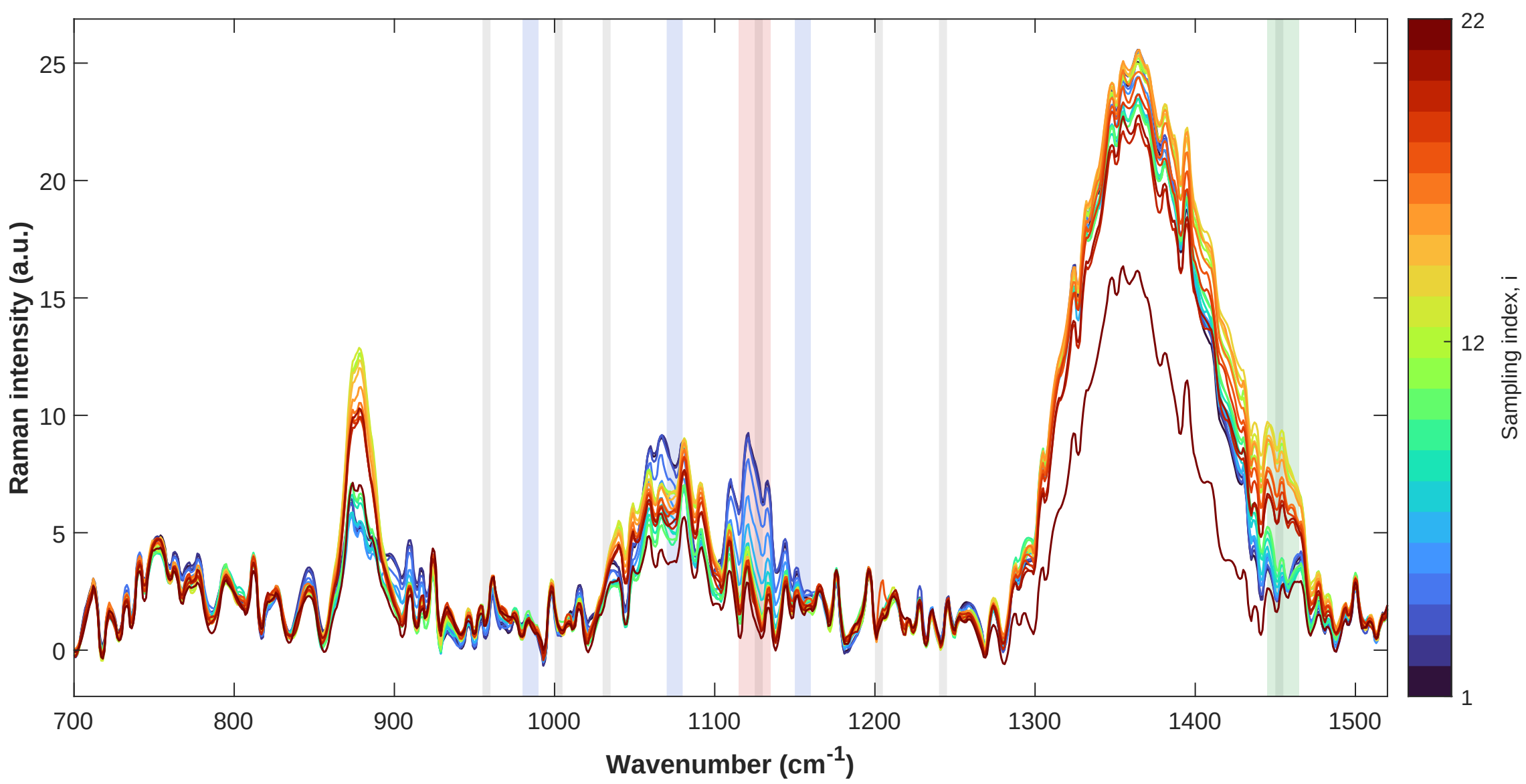

Figure S1: Overview of all 22 airPLS-corrected mean Raman spectra used as candidate inputs for sample subset selection on F1. Each line represents the pointwise mean of 110 consecutive in-line Raman acquisitions associated with one candidate offline sampling time. Line colours indicate the sampling index i. The coloured vertical bands denote the literature-derived wavenumber regions used for the analyte-specific WS feature representations: grey for biomass-related bands, red for the glucose-related band, green for the ammonium-related band, and blue for phosphate-related bands. The spectra are displayed from 700 to 1520 cm^{-1}. These coloured bands denote feature regions and do not represent an interquartile range, standard deviation, or other statistical uncertainty.

Figures S2–S4 show the individual acquisitions for the remaining 17 candidate sampling windows not displayed in the main manuscript. Thin grey lines denote the individual acquisitions, black lines denote the corresponding pointwise mean spectra, and grey envelopes indicate the mean ± one standard deviation calculated separately at each wavenumber.

A comparison between the mean and median spectra showed close agreement across the 22 sampling windows. The RMSE between the mean and median spectrum, normalized by the peak-to-peak range of the corresponding mean spectrum, had a median value of 0.00574 and a maximum value of 0.02675. The mean representation was therefore retained consistently with the original CVQ- and KS-based subset-selection workflow.

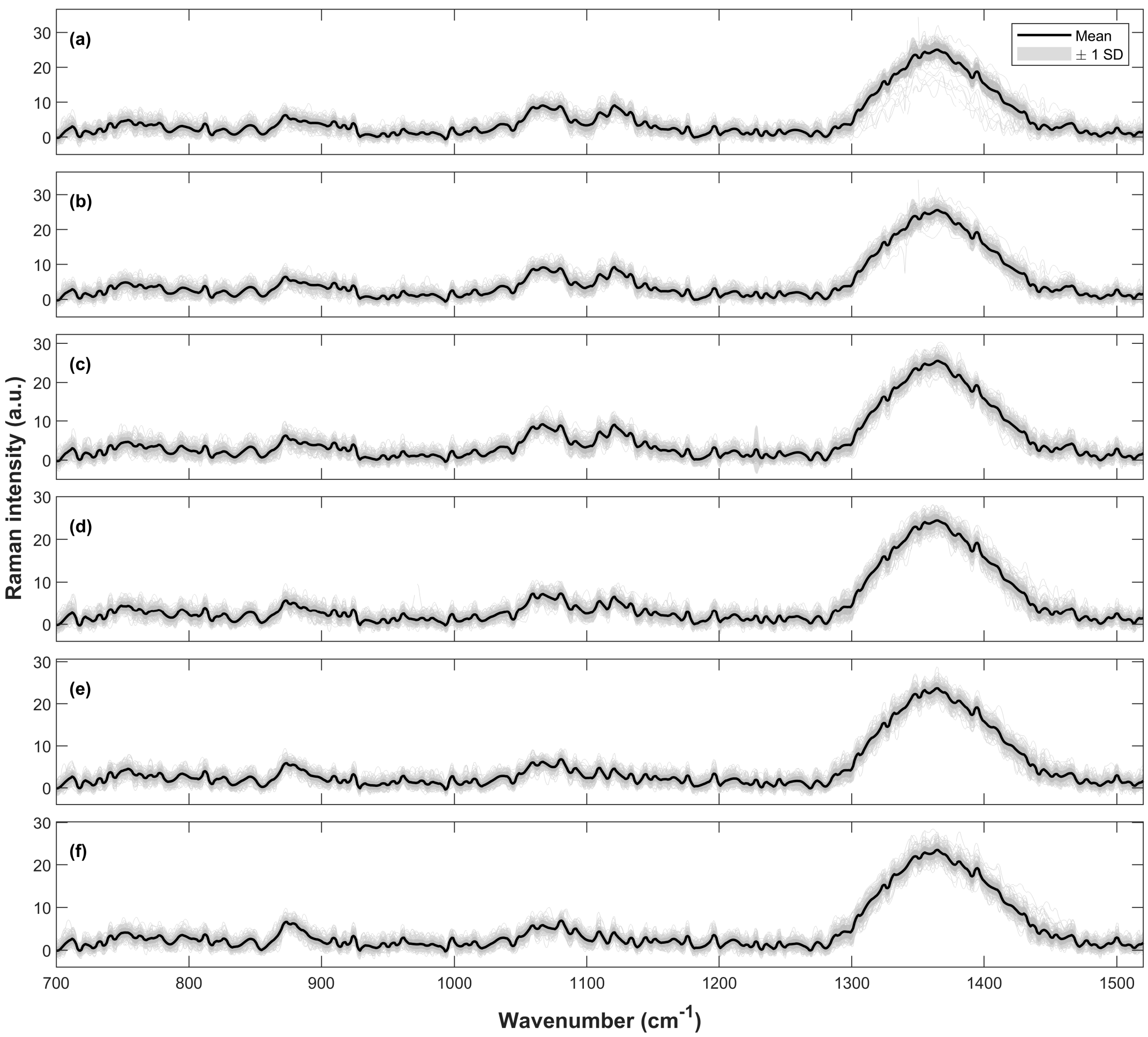

Figure S2: Within-window variability of the airPLS-corrected in-line Raman spectra for candidate sampling indices 1, 2, 3, 5, 7, and 8. Panels (a)–(f) correspond to these sampling indices in the stated order. Each panel shows all 110 consecutive Raman acquisitions recorded during the associated approximately 10-min sampling window. Thin grey lines denote the individual acquisitions, black lines denote the pointwise mean spectra, and grey envelopes indicate the mean ± one standard deviation calculated separately at each wavenumber.

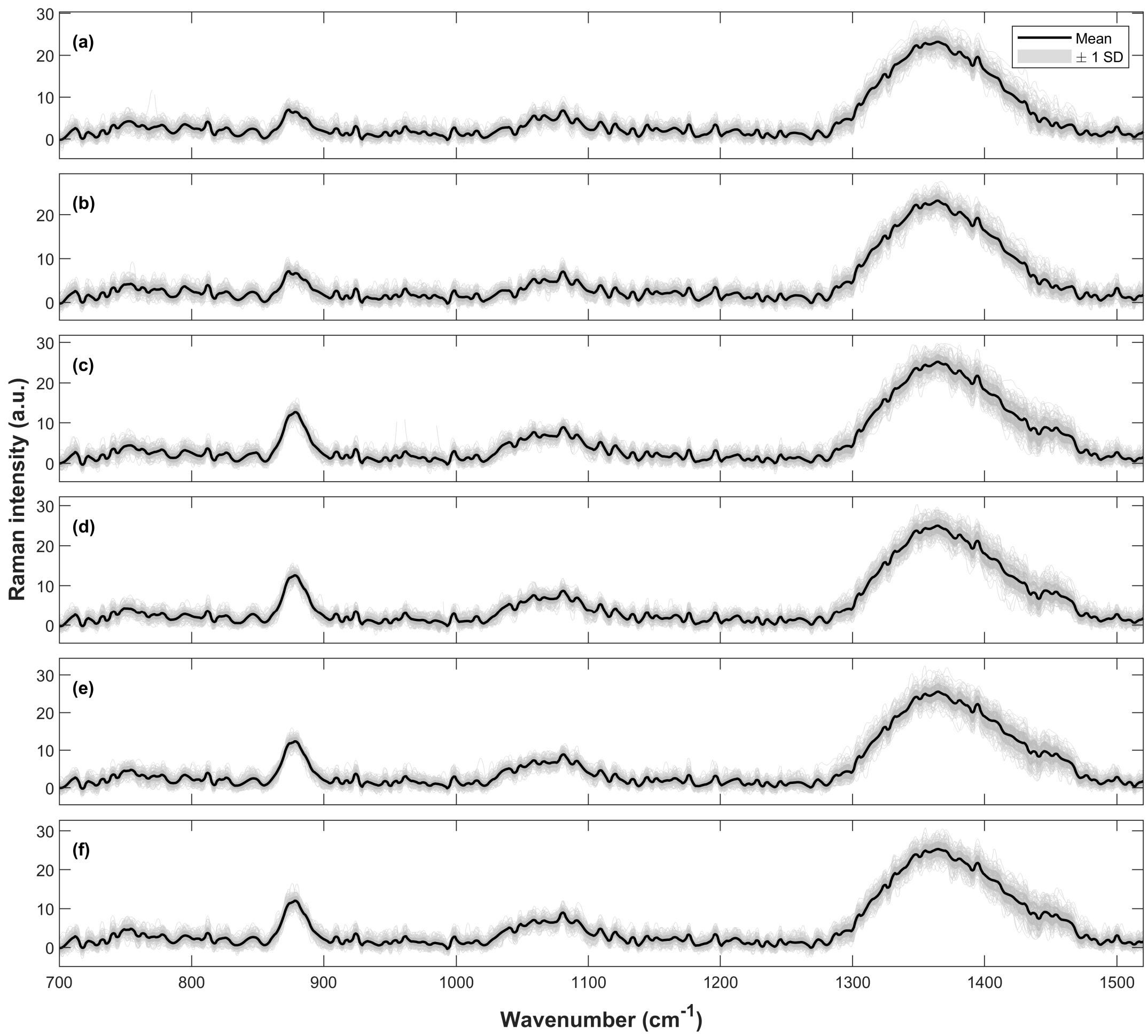

Figure S3: Within-window variability of the airPLS-corrected in-line Raman spectra for candidate sampling indices 9, 10, 11, 12, 14, and 15. Panels (a)–(f) correspond to these sampling indices in the stated order. Each panel shows all 110 consecutive Raman acquisitions. Thin grey lines denote the individual acquisitions, black lines denote the pointwise mean spectra, and grey envelopes indicate the mean ± one standard deviation calculated separately at each wavenumber.

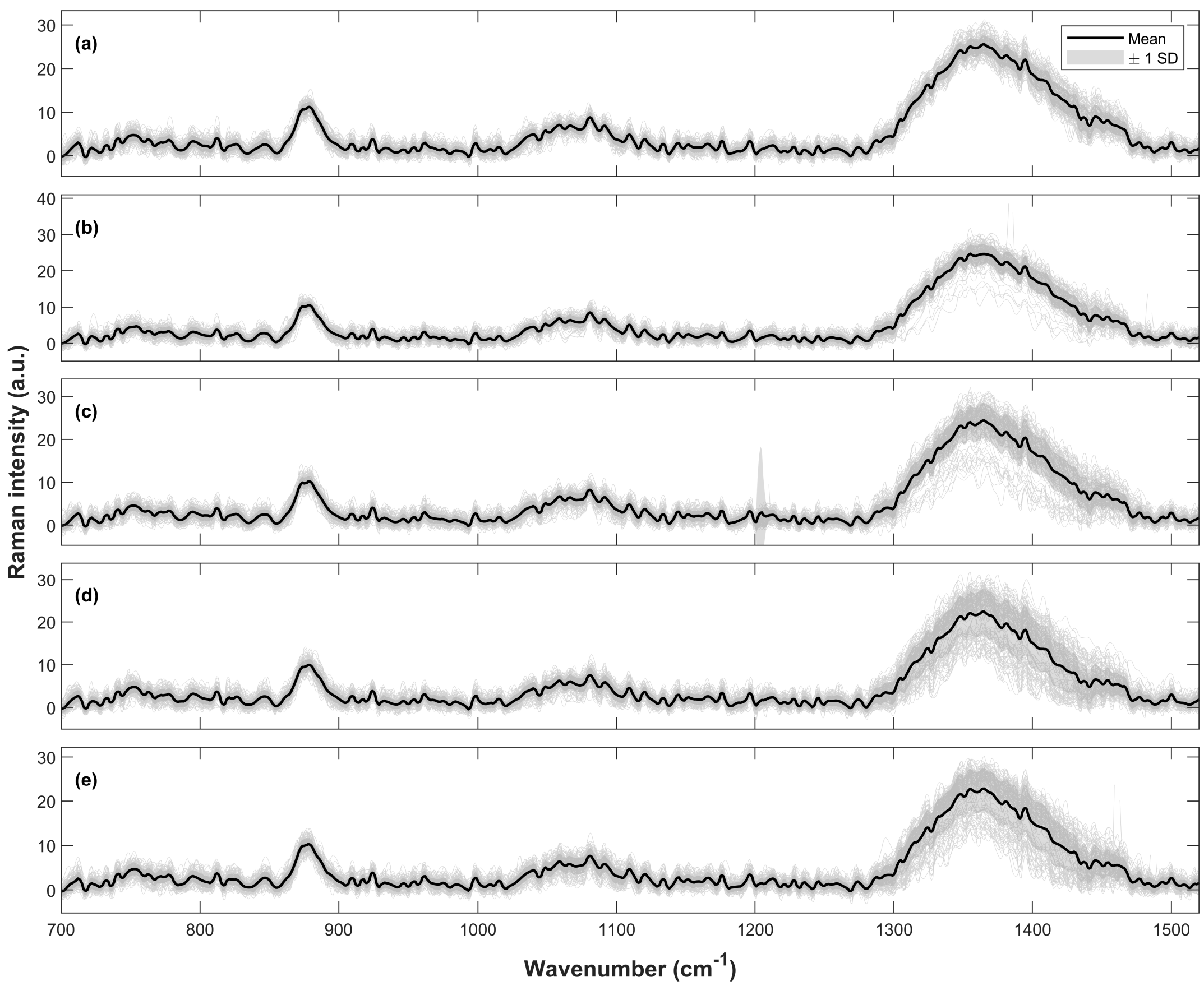

Figure S4: Within-window variability of the airPLS-corrected in-line Raman spectra for candidate sampling indices 16, 17, 18, 20, and 21. Panels (a)–(e) correspond to these sampling indices in the stated order. Each panel shows all 110 consecutive Raman acquisitions. Thin grey lines denote the individual acquisitions, black lines denote the pointwise mean spectra, and grey envelopes indicate the mean ± one standard deviation calculated separately at each wavenumber.

1.2 Parameter Identification

The following graphical notation is used in Figs. S5–S13: selected offline reference samples are shown as black markers with error bars, available but non-selected offline samples are shown as green markers, online measurements and feeding rates are shown as thick blue lines, solid black lines indicate model simulations obtained with the reduced sampling strategy named in the respective caption, and dashed black lines indicate the full-data reference (FD) simulation. Error bars denote the experimental measurement uncertainty used for the corresponding offline reference data.

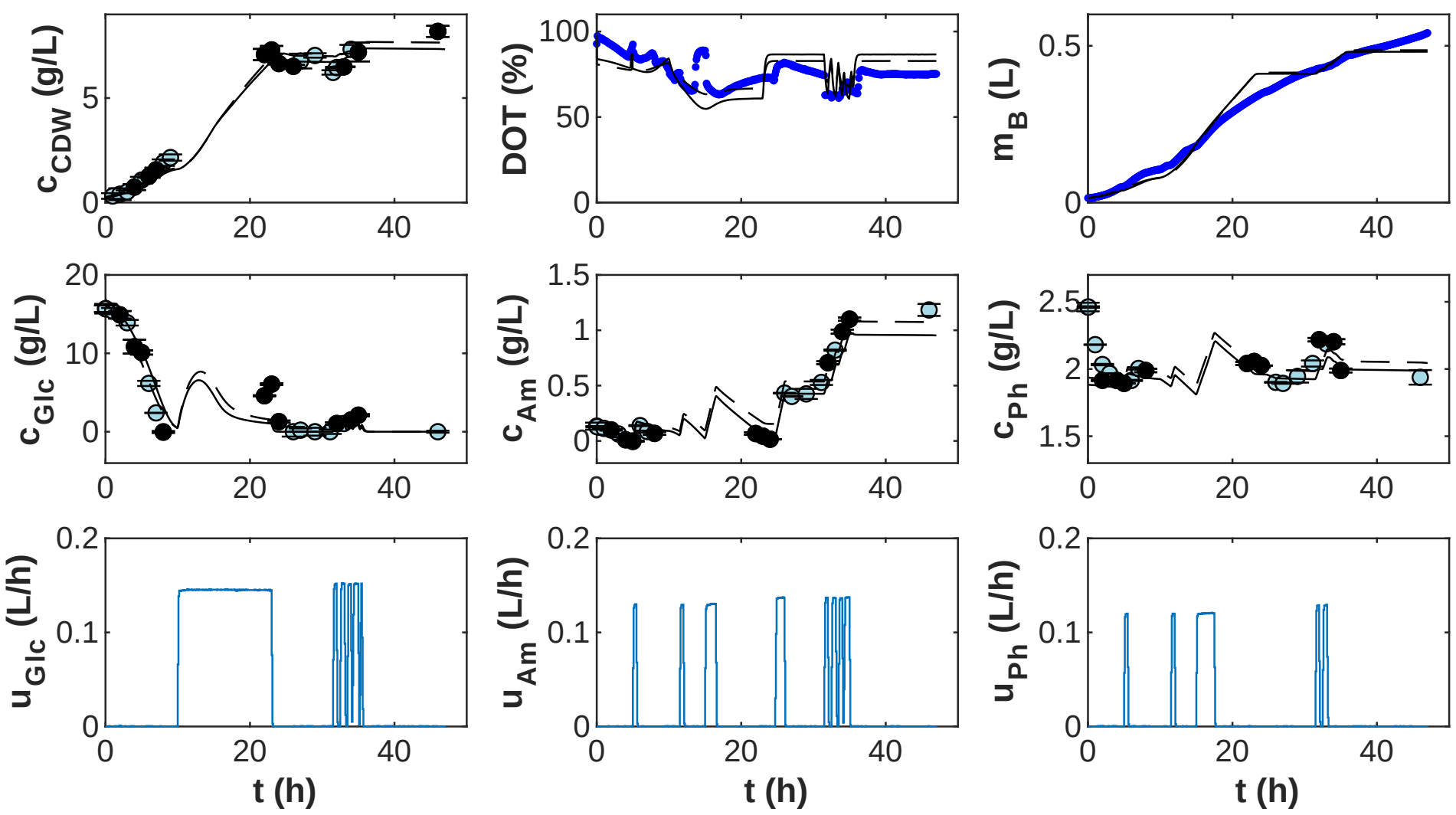

Figure S5: PI auto-validation (F1) using FS-CVQ-10. Selected offline reference samples are shown as black markers with error bars, whereas available but non-selected offline samples are shown as green markers. Online measurements and feeding rates are shown as thick blue lines. The solid black lines represent the PI simulation obtained with FS-CVQ-10, whereas dashed black lines represent the full-data reference (FD) simulation.

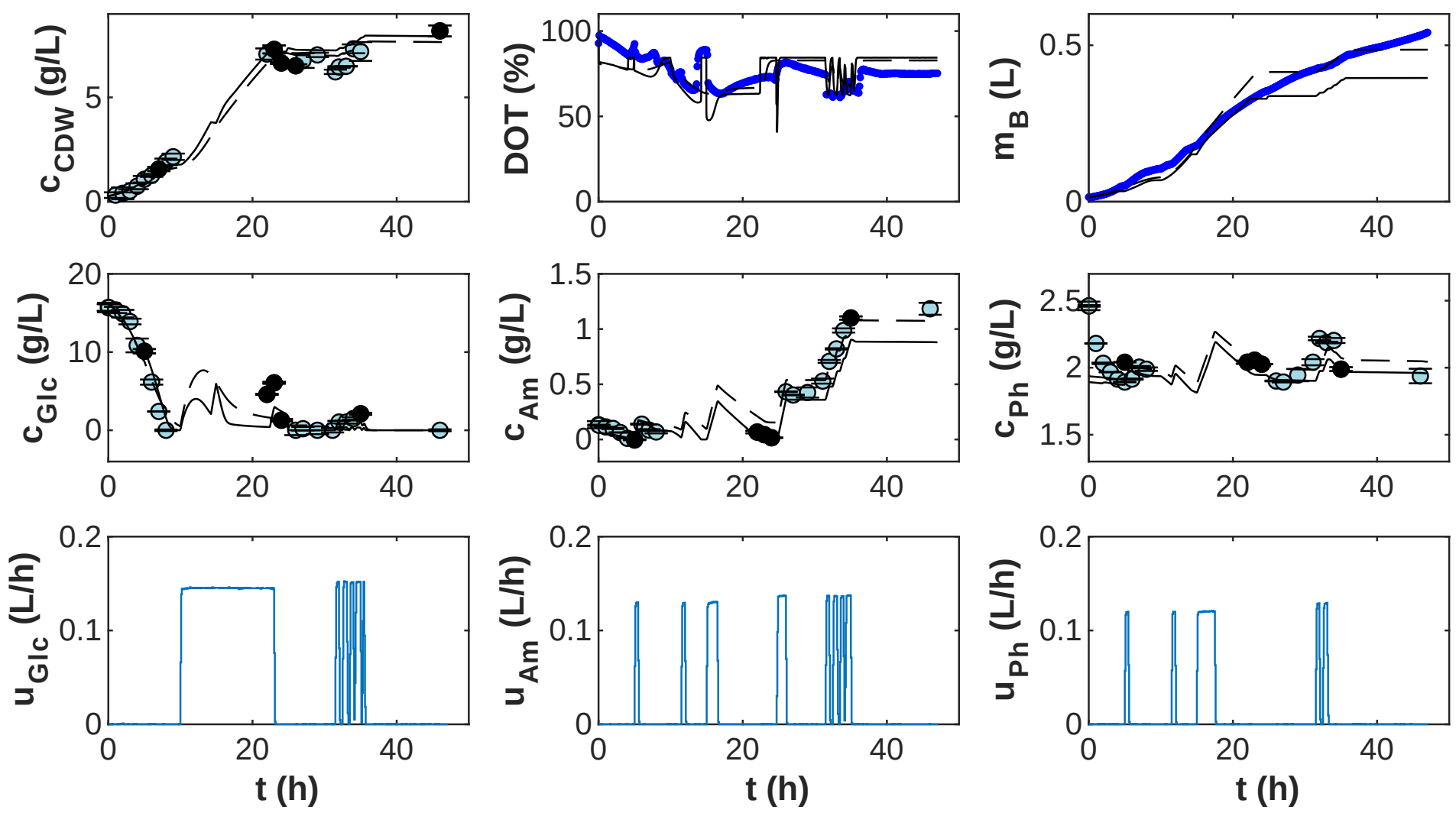

Figure S6: PI auto-validation (F1) using FS-CVQ-05. Selected offline reference samples are shown as black markers with error bars, whereas available but non-selected offline samples are shown as green markers. Online measurements and feeding rates are shown as thick blue lines. The solid black lines represent the PI simulation obtained with FS-CVQ-05, whereas dashed black lines represent the full-data reference (FD) simulation.

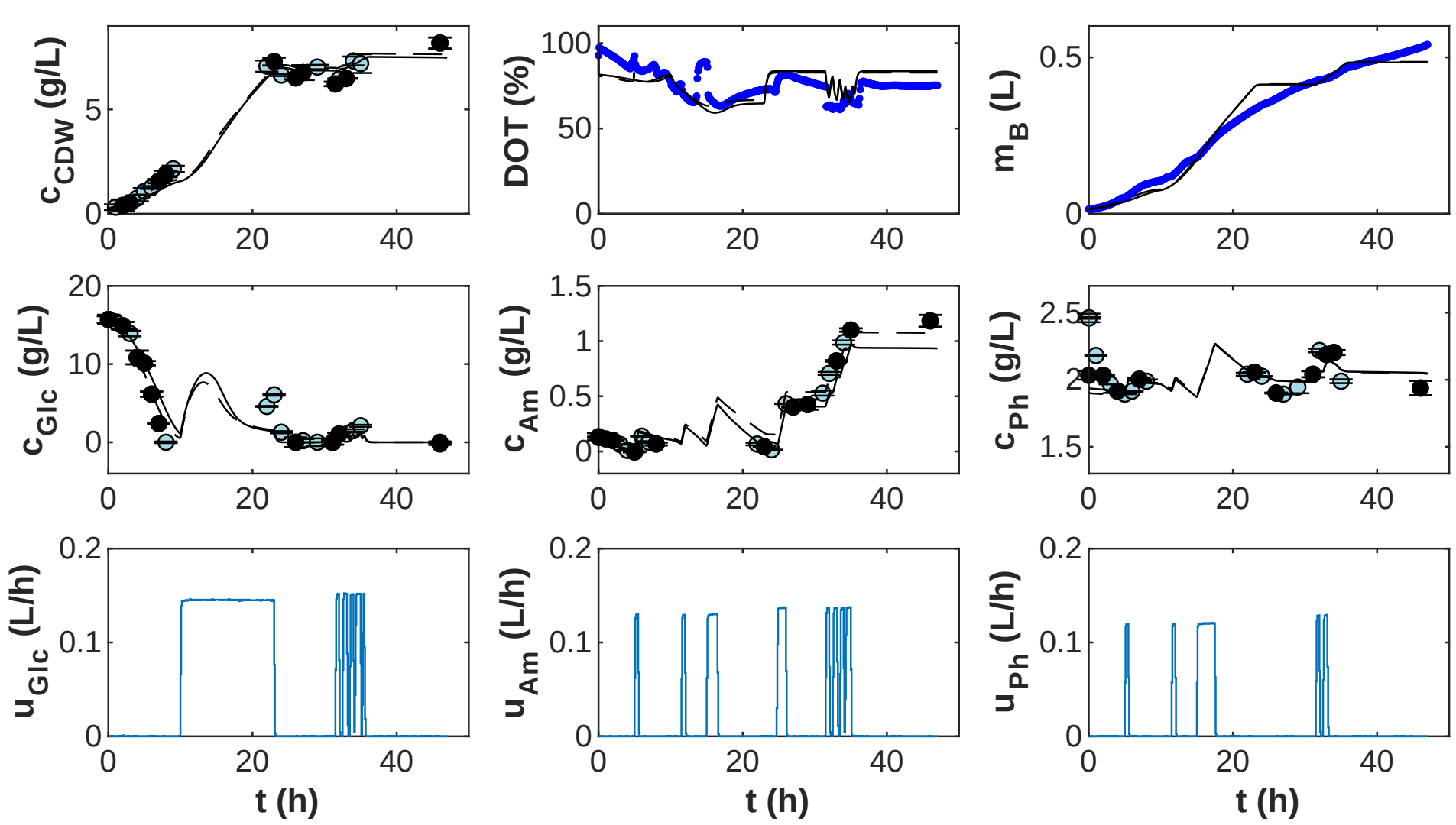

Figure S7: PI auto-validation (F1) using WS-CVQ-10. Selected offline reference samples are shown as black markers with error bars, whereas available but non-selected offline samples are shown as green markers. Online measurements and feeding rates are shown as thick blue lines. The solid black lines represent the PI simulation obtained with WS-CVQ-10, whereas dashed black lines represent the full-data reference (FD) simulation.

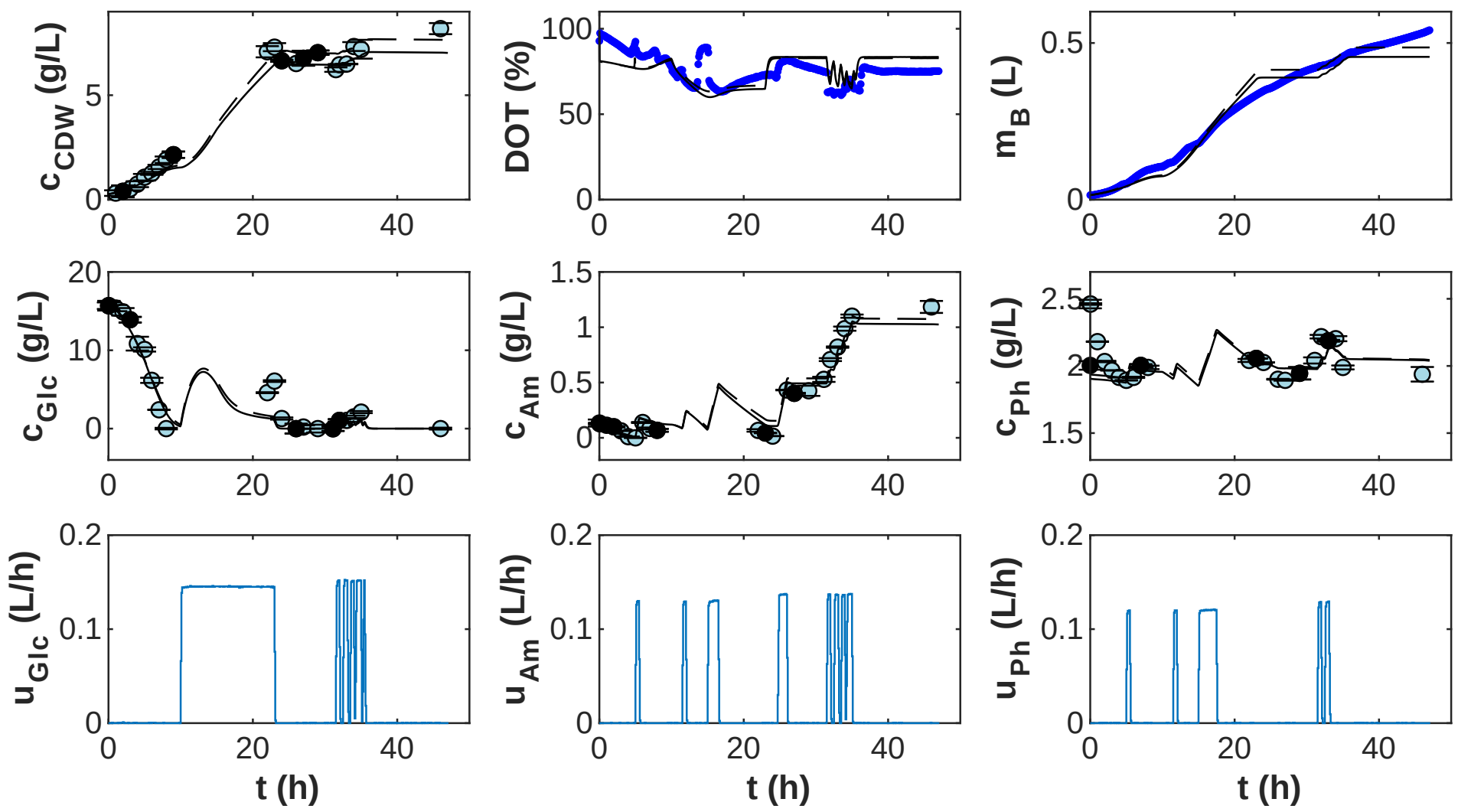

Figure S8: PI auto-validation (F1) using WS-CVQ-05. Selected offline reference samples are shown as black markers with error bars, whereas available but non-selected offline samples are shown as green markers. Online measurements and feeding rates are shown as thick blue lines. The solid black lines represent the PI simulation obtained with WS-CVQ-05, whereas dashed black lines represent the full-data reference (FD) simulation.

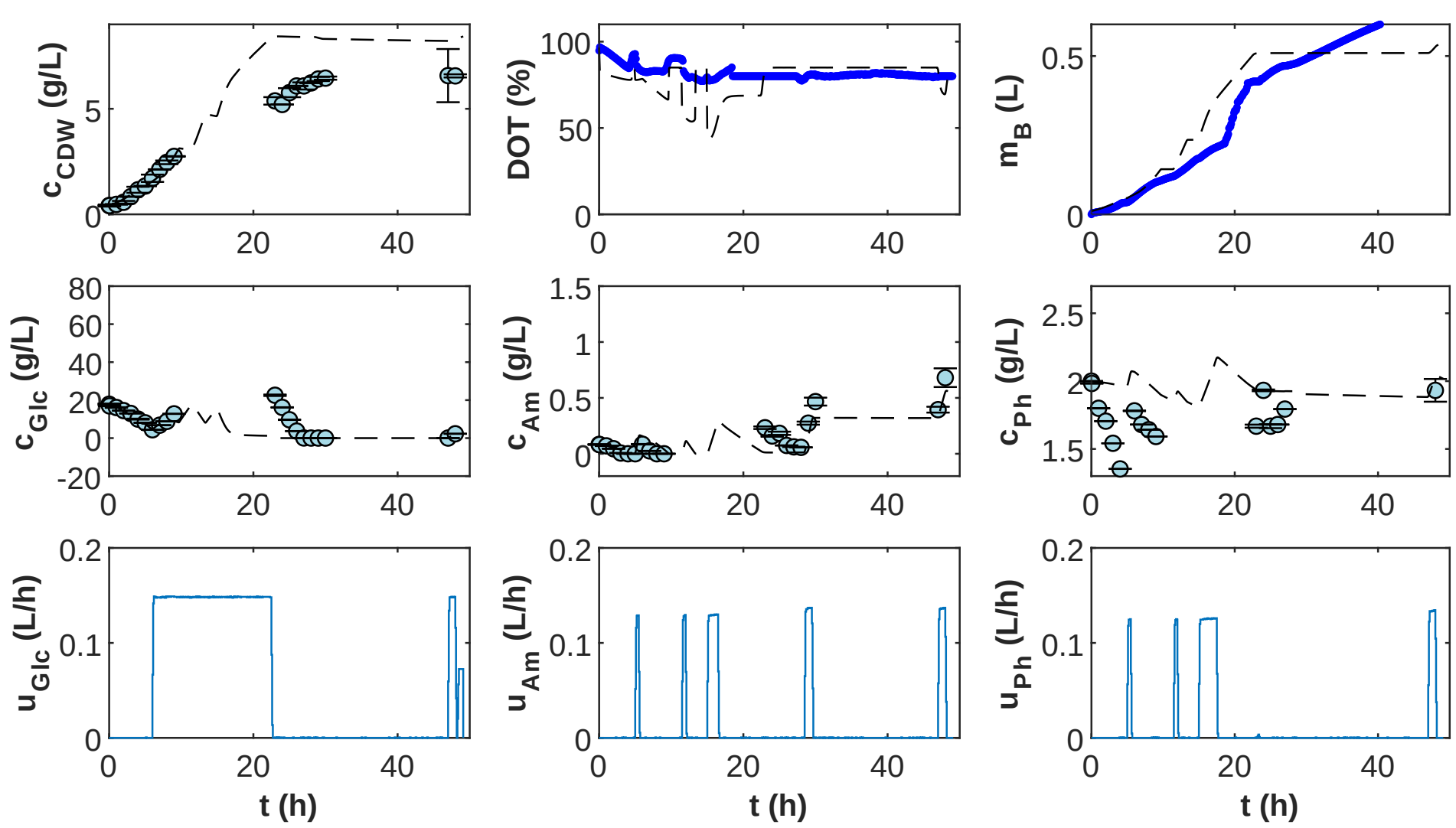

Figure S9: Cross-validation (F2) using the full-data reference (FD). Offline reference samples are shown as green markers with error bars. Online measurements and feeding rates are shown as thick blue lines. The dashed black lines represent the PI cross-validation simulation obtained with the FD parameter set.

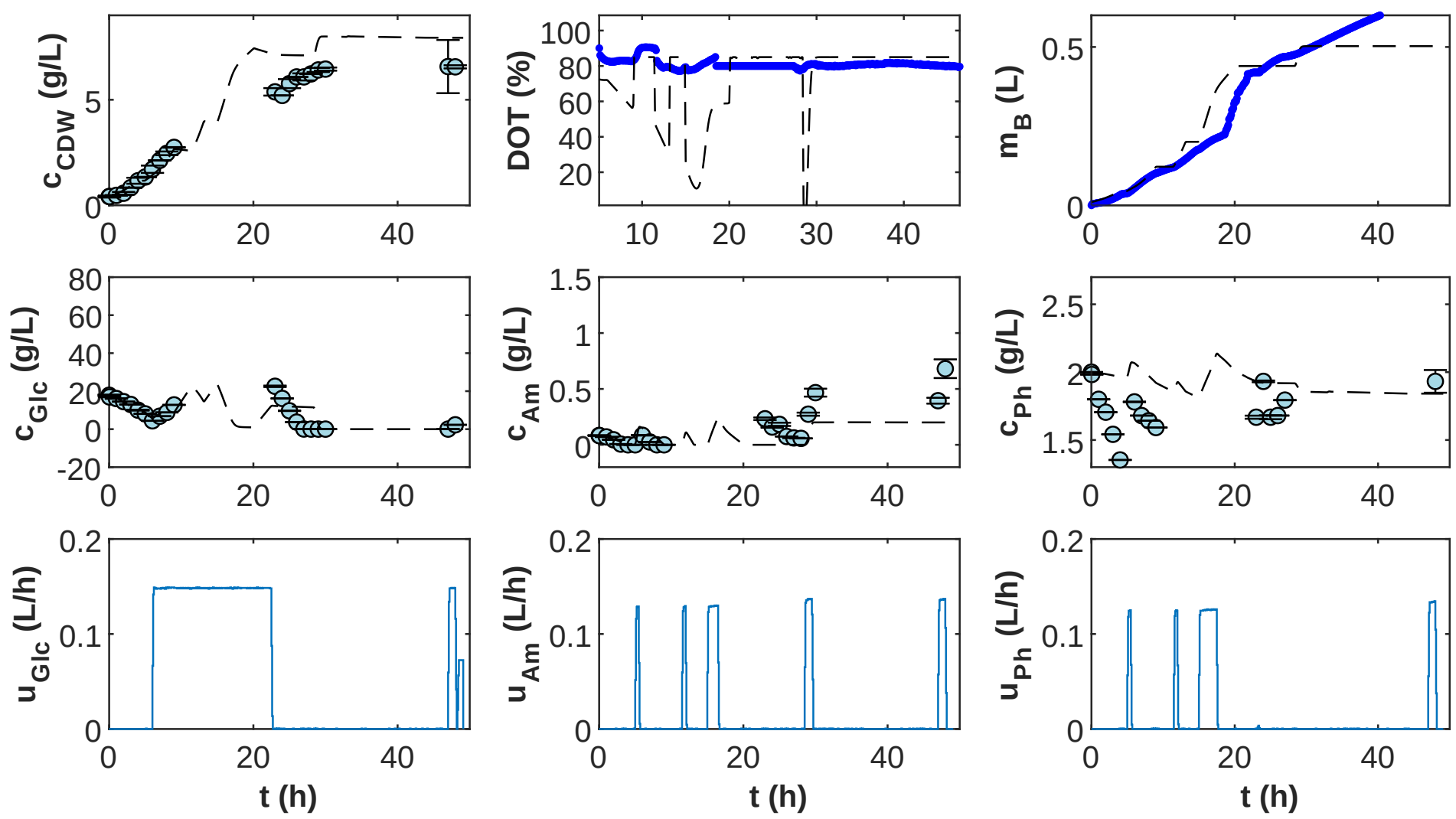

Figure S10: Cross-validation (F2) using FS-CVQ-10. Offline reference samples are shown as green markers with error bars. Online measurements and feeding rates are shown as thick blue lines. The dashed black lines represent the PI cross-validation simulation obtained with the FS-CVQ-10 parameter set.

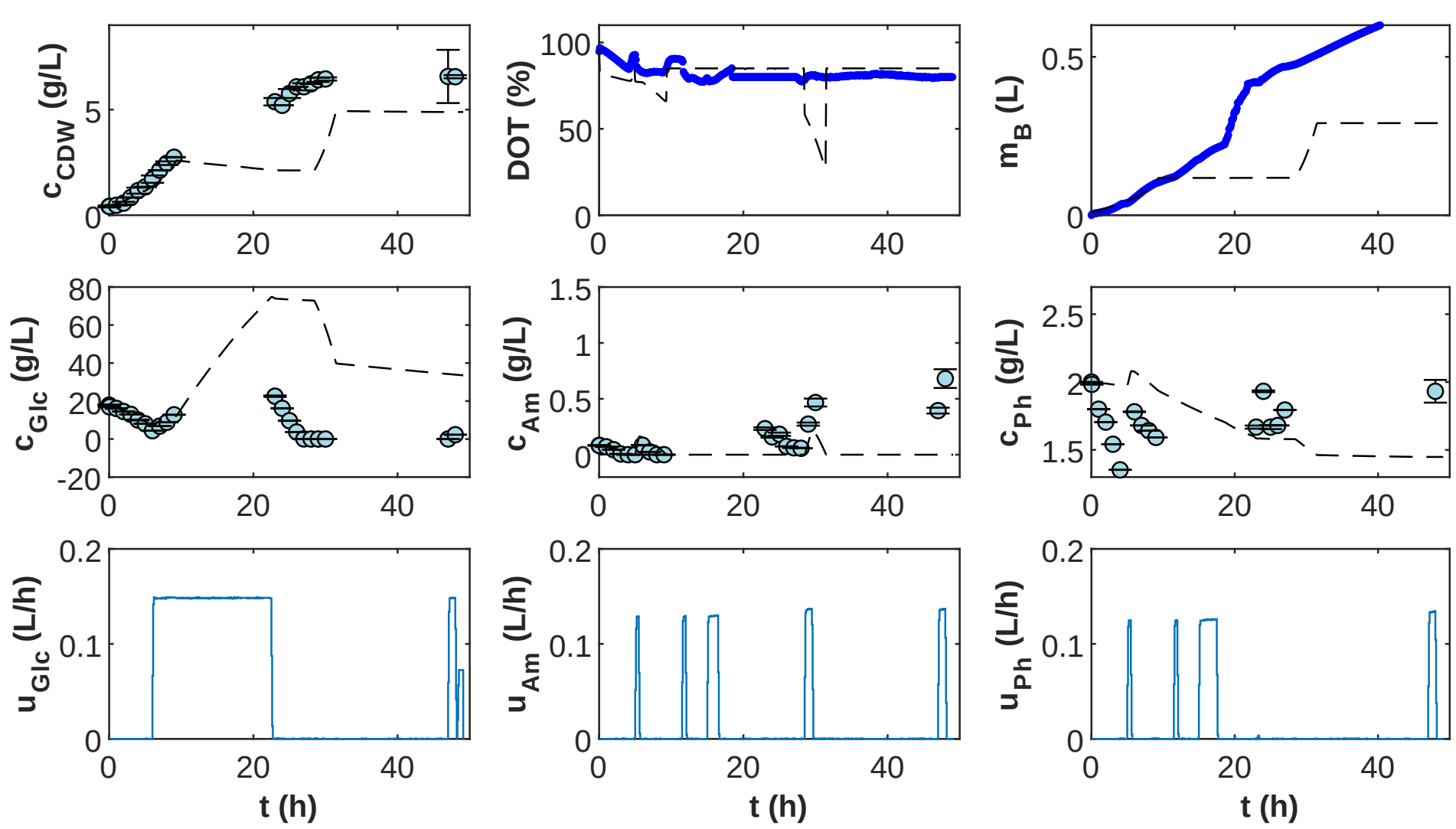

Figure S11: Cross-validation (F2) using FS-CVQ-05. Offline reference samples are shown as green markers with error bars. Online measurements and feeding rates are shown as thick blue lines. The dashed black lines represent the PI cross-validation simulation obtained with the FS-CVQ-05 parameter set.

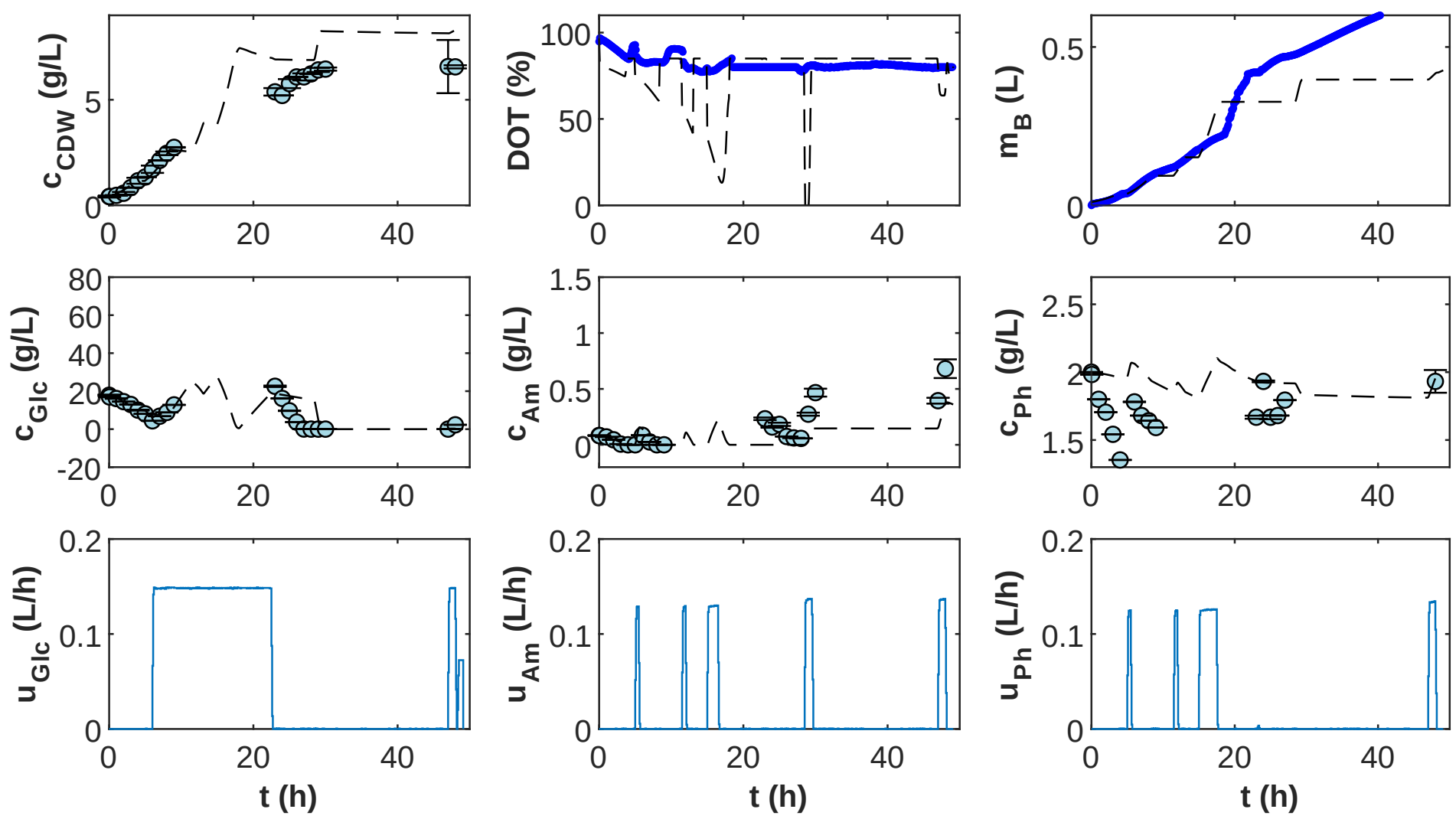

Figure S12: Cross-validation (F2) using WS-CVQ-10. Offline reference samples are shown as green markers with error bars. Online measurements and feeding rates are shown as thick blue lines. The dashed black lines represent the PI cross-validation simulation obtained with the WS-CVQ-10 parameter set.

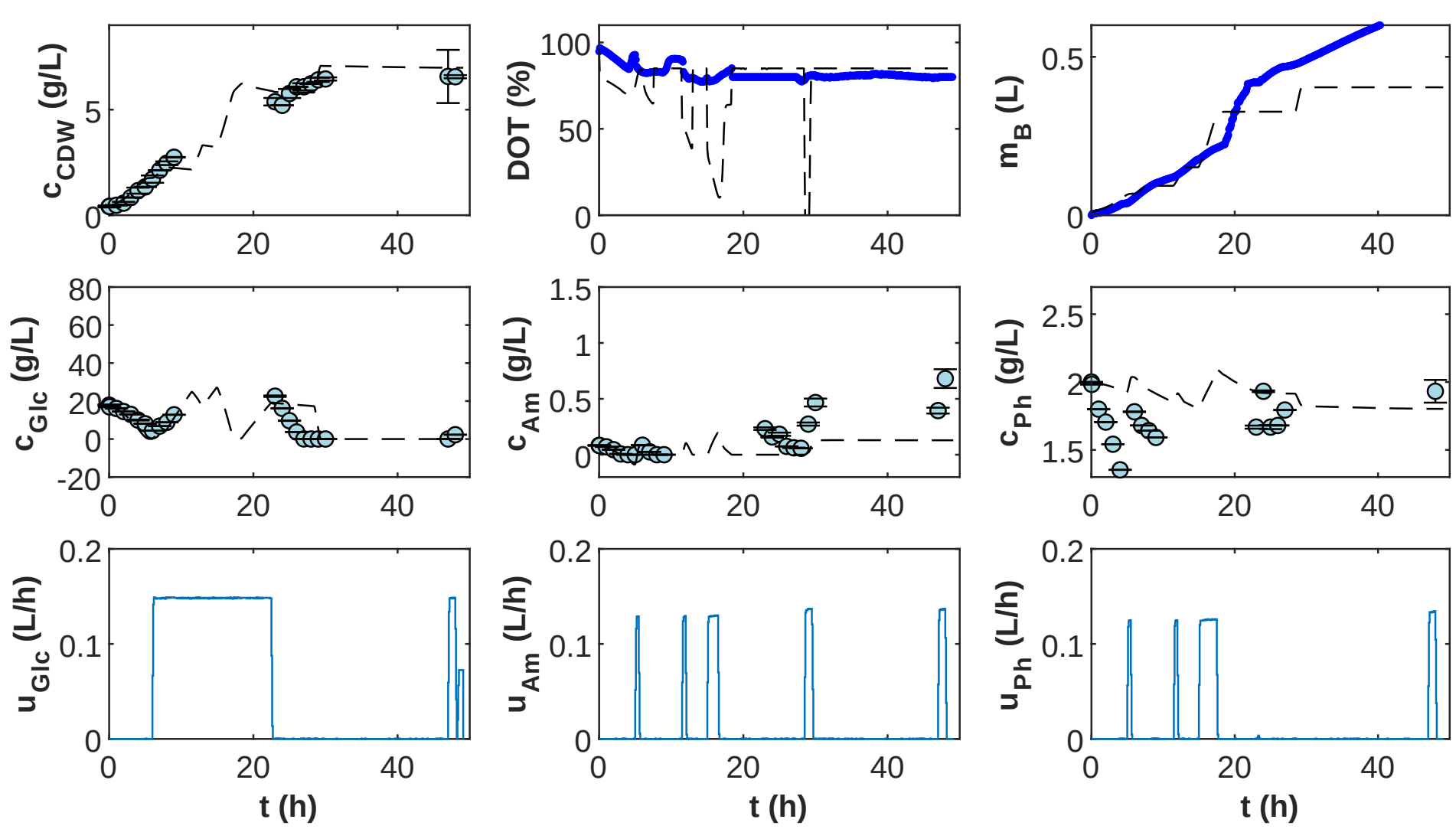

Figure S13: Cross-validation (F2) using WS-CVQ-05. Offline reference samples are shown as green markers with error bars. Online measurements and feeding rates are shown as thick blue lines. The dashed black lines represent the PI cross-validation simulation obtained with the WS-CVQ-05 parameter set.

2 Supplementary Tables

Table S1: States $\boldsymbol{x}$, measurements $\boldsymbol{y}$, inputs $\boldsymbol{u}$, parameters $\boldsymbol{\theta}$, and constants.

**Symbol**	**Unit**	**Type**	**Description**
m_{CDW}	g	state	Biomass mass (cell dry weight)
m_{Glc}	g	state	Glucose mass
m_{Am}	g	state	Ammonium mass
m_{Ph}	g	state	Phosphate mass
DOT	%	state	Dissolved oxygen tension
m_{B}	L	state	Total volume of consumed base
V	L	state	Broth volume
$c_i = m_i/V$	$\mathrm{g\,L^{-1}}$	meas.	Concentrations ($i \in \{\mathrm{CDW}, \mathrm{Glc}, \mathrm{Am}, \mathrm{Ph}\}$)
$\boldsymbol{y} = [c_{\mathrm{CDW}}, c_{\mathrm{Glc}}, c_{\mathrm{Am}}, c_{\mathrm{Ph}}, DOT, m_B]^\top$	–	meas.	Measurement vector
$u_{\mathrm{Glc}}, u_{\mathrm{Am}}, u_{\mathrm{Ph}}, u_B, u_A, u_{\mathrm{sample}}$	$\mathrm{L\,h^{-1}}$	input	Feed/base/acid/sample flow rates
$c_{\mathrm{Glc,feed}}, c_{\mathrm{Am,feed}}, c_{\mathrm{Ph,feed}}$	$\mathrm{g\,L^{-1}}$	const.	Substrate concentrations in feed
r_{CDW}	$\mathrm{h^{-1}}$	rate	Specific growth rate
r_{Glc}	$\mathrm{h^{-1}}$	rate	Substrate limitation term
r_{Maint}	$\mathrm{h^{-1}}$	rate	Maintenance term
$\mu_{\mathrm{Glc,max}}$	$\mathrm{h^{-1}}$	param	Max. specific growth rate
$\mu_{\mathrm{Maint,max}}$	$\mathrm{h^{-1}}$	param	Max. specific maintenance rate
$Y_{\mathrm{Glc/CDW}}$	$\mathrm{g\,g^{-1}}$	param	Glucose consumption per biomass produced
$Y_{\mathrm{Am/CDW}}$	$\mathrm{g\,g^{-1}}$	param	Ammonium consumption per biomass produced
$Y_{\mathrm{Ph/CDW}}$	$\mathrm{g\,g^{-1}}$	param	Phosphate consumption per biomass produced
$Y_{\mathrm{O/Glc}}$	$\mathrm{g\,g^{-1}}$	param	Oxygen consumed per glucose utilized
$Y_{\mathrm{B/Am}}$	$\mathrm{g\,g^{-1}}$	param	Base formation per ammonium consumed
$k_{\mathrm{m,Glc}}$	$\mathrm{g\,L^{-1}}$	param	Monod constant for glucose uptake
$k_{\mathrm{m,Maint}}$	$\mathrm{g\,L^{-1}}$	param	Monod constant for maintenance metabolism
$k_{\mathrm{L}}a$	$\mathrm{h^{-1}}$	param	Volumetric oxygen transfer coefficient
DOT^*	% air sat.	const.	Gas–liquid equilibrium DOT (calibrated as 100%)
H	$\mathrm{\%\,L\,g^{-1}}$	const.	Henry constant
$\boldsymbol{\theta}$	–	param	Parameter vector
Φ_{PI}	–	cost	PI objective (modified MLE)
$W_{k,l}$	–	weight	Optional weighting for sampling heterogeneity
$\tilde{C}_{k,l}$	–	cov.	Measurement covariance used in PI

Table S2: Selected sampling time points for all deterministic sample subset-selection strategies and analytes. UI and FS-based methods use one common subset for all analytes. WS-based methods select n_{sel} reference assays per analyte and can therefore use different sampling time points for different analytes. Initial model values at BA = 0 are used only for model initialization and are not counted as candidate offline samples.

**Strategy**	**Analyte**	**Selected t_M (h)**
FD	CDW	0.5, 1, 1.5, 2, 3, 4, 5, 6, 7, 8, 22, 23, 24, 26, 27, 29, 31, 32, 33, 34, 35, 46
	Glc	0.5, 1, 1.5, 2, 3, 4, 5, 6, 7, 8, 22, 23, 24, 26, 27, 29, 31, 32, 33, 34, 35, 46
	Am	0.5, 1, 1.5, 2, 3, 4, 5, 6, 7, 8, 22, 23, 24, 26, 27, 29, 31, 32, 33, 34, 35, 46
	Ph	0.5, 1, 1.5, 2, 3, 4, 5, 6, 7, 8, 22, 23, 24, 26, 27, 29, 31, 32, 33, 34, 35, 46
UI-05	CDW	0.5, 8, 23, 34, 46
	Glc	0.5, 8, 23, 34, 46
	Am	0.5, 8, 23, 34, 46
	Ph	0.5, 8, 23, 34, 46
UI-10	CDW	0.5, 5, 8, 22, 23, 26, 31, 34, 35, 46
	Glc	0.5, 5, 8, 22, 23, 26, 31, 34, 35, 46
	Am	0.5, 5, 8, 22, 23, 26, 31, 34, 35, 46
	Ph	0.5, 5, 8, 22, 23, 26, 31, 34, 35, 46
FS-CVQ-05	CDW	5, 22, 23, 24, 35
	Glc	5, 22, 23, 24, 35
	Am	5, 22, 23, 24, 35
	Ph	5, 22, 23, 24, 35
FS-CVQ-10	CDW	3, 5, 6, 22, 23, 24, 26, 33, 35, 46
	Glc	3, 5, 6, 22, 23, 24, 26, 33, 35, 46
	Am	3, 5, 6, 22, 23, 24, 26, 33, 35, 46
	Ph	3, 5, 6, 22, 23, 24, 26, 33, 35, 46
WS-CVQ-05	CDW	0.5, 7, 23, 26, 27
	Glc	0.5, 3, 26, 31, 32
	Am	0.5, 2, 8, 23, 27
	Ph	0.5, 7, 23, 29, 33
WS-CVQ-10	CDW	0.5, 2, 6, 7, 23, 26, 27, 31, 33, 46
	Glc	0.5, 2, 4, 5, 6, 7, 26, 31, 32, 46
	Am	0.5, 2, 5, 8, 23, 27, 29, 33, 35, 46
	Ph	0.5, 2, 4, 7, 23, 26, 31, 33, 34, 46
FS-KS-05	CDW	0.5, 8, 29, 34, 46
	Glc	0.5, 8, 29, 34, 46
	Am	0.5, 8, 29, 34, 46
	Ph	0.5, 8, 29, 34, 46
FS-KS-10	CDW	0.5, 3, 5, 8, 22, 23, 29, 33, 34, 46
	Glc	0.5, 3, 5, 8, 22, 23, 29, 33, 34, 46
	Am	0.5, 3, 5, 8, 22, 23, 29, 33, 34, 46
	Ph	0.5, 3, 5, 8, 22, 23, 29, 33, 34, 46
WS-KS-05	CDW	0.5, 7, 29, 34, 46
	Glc	0.5, 3, 7, 24, 46
	Am	8, 24, 31, 35, 46
	Ph	0.5, 3, 7, 27, 46
WS-KS-10	CDW	0.5, 2, 3, 4, 7, 22, 29, 33, 34, 46
	Glc	0.5, 2, 3, 4, 5, 7, 24, 31, 34, 46
	Am	4, 8, 23, 24, 26, 31, 33, 34, 35, 46
	Ph	0.5, 2, 3, 4, 7, 22, 27, 29, 35, 46

Table S3: Union of selected physical sampling time points for deterministic subset-selection strategies. UI and FS-based methods use one common subset for all analytes. WS-based methods select n_{sel} reference assays per analyte; consequently, the union of unique physical sampling time points can be larger than n_{sel}.

**Strategy**	**Number of unique t_M**	**Union of selected t_M (h)**
UI-05	5	0.5, 8, 23, 34, 46
UI-10	10	0.5, 5, 8, 22, 23, 26, 31, 34, 35, 46
FS-CVQ-05	5	5, 22, 23, 24, 35
FS-CVQ-10	10	3, 5, 6, 22, 23, 24, 26, 33, 35, 46
FS-KS-05	5	0.5, 8, 29, 34, 46
FS-KS-10	10	0.5, 3, 5, 8, 22, 23, 29, 33, 34, 46
WS-CVQ-05	13	0.5, 1, 2, 3, 7, 8, 23, 26, 27, 29, 31, 32, 33
WS-CVQ-10	18	0.5, 1, 2, 4, 5, 6, 7, 8, 23, 26, 27, 29, 31, 32, 33, 34, 35, 46
WS-KS-05	12	0.5, 1, 3, 7, 8, 24, 27, 29, 31, 34, 35, 46
WS-KS-10	19	0.5, 1, 2, 3, 4, 5, 7, 8, 22, 23, 24, 26, 27, 29, 31, 33, 34, 35, 46

Table S4: Selected sampling time points for the random subsampling baseline RS-05.

**Run**	**Selected t_M (h)**
RS-05 run 01	0.5, 1, 4, 8, 29
RS-05 run 02	0.5, 7, 24, 27, 31
RS-05 run 03	0.5, 2, 6, 8, 26
RS-05 run 04	0.5, 2, 4, 22, 29
RS-05 run 05	3, 31, 32, 33, 46
RS-05 run 06	0.5, 4, 23, 31, 32
RS-05 run 07	0.5, 2, 3, 5, 33
RS-05 run 08	0.5, 3, 5, 22, 23
RS-05 run 09	0.5, 2, 5, 23, 27
RS-05 run 10	2, 22, 27, 29, 32
RS-05 run 11	1, 3, 4, 29, 46
RS-05 run 12	0.5, 6, 7, 8, 22
RS-05 run 13	0.5, 24, 29, 32, 33
RS-05 run 14	4, 8, 24, 27, 31
RS-05 run 15	23, 24, 26, 34, 46
RS-05 run 16	3, 7, 32, 33, 46
RS-05 run 17	0.5 1, 5, 31, 34
RS-05 run 18	0.5, 7, 26, 31, 32
RS-05 run 19	3, 22, 32, 34, 35
RS-05 run 20	0.5, 1, 8, 22, 33

Table S5: Selected sampling time points for the random subsampling baseline RS-10.

**Run**	**Selected t_M (h)**
RS-10 run 01	0.5, 1, 2, 5, 6, 8, 24, 26, 32, 33
RS-10 run 02	0.5 1, 1.5, 2, 4, 6, 7, 8, 24, 27
RS-10 run 03	1, 3, 5, 6, 7, 23, 24, 31, 34, 46
RS-10 run 04	0.5, 1, 2, 3, 22, 26, 31, 32, 33, 34
RS-10 run 05	0.5 1, 1.5, 6, 24, 26, 32, 33, 34, 46
RS-10 run 06	1, 4, 22, 23, 26, 27, 29, 31, 32, 33
RS-10 run 07	0.5, 1, 4, 5, 7, 22, 26, 29, 32, 35, 46
RS-10 run 08	1, 2, 3, 4, 5, 24, 27, 32, 33, 34
RS-10 run 09	0.5, 1, 2, 5, 6, 8, 24, 26, 32, 33
RS-10 run 10	0.5 1, 3, 5, 6, 26, 31, 32, 34, 35
RS-10 run 11	0.5, 4, 7, 22, 23, 26, 29, 31, 33, 34
RS-10 run 12	0.5, 1, 7, 8, 22, 24, 26, 27, 29, 46
RS-10 run 13	0.5, 1, 2, 3, 4, 7, 8, 22, 32, 46
RS-10 run 14	0.5, 1, 1.5, 5, 6, 7, 8, 24, 29, 31, 33
RS-10 run 15	3, 8, 23, 27, 29, 31, 33, 34, 35, 46
RS-10 run 16	0.5 1, 2, 5, 6, 7, 22, 31, 33, 35
RS-10 run 17	2, 5, 6, 8, 22, 23, 26, 31, 34, 46
RS-10 run 18	0.5, 1, 3, 8, 22, 24, 26, 27, 29, 46
RS-10 run 19	2, 5, 6, 7, 22, 24, 29, 32, 33, 35
RS-10 run 20	0.5, 1, 2, 4, 5, 6, 27, 31, 34, 46

The pointwise mean spectrum was compared with the corresponding pointwise median spectrum for each of the 22 sampling windows. The RMSE was normalized by the peak-to-peak range of the respective mean spectrum. As summarized in Table S6, the median range-normalized RMSE was 0.574%, and the maximum range-normalized RMSE was 2.675%.

Table S6: Agreement between the pointwise mean and median airPLS-corrected Raman spectra for the 22 candidate sampling windows. RMSE was calculated across the analysed spectral range. The range-normalized RMSE between the mean and median spectra was obtained by normalizing the RMSE by the peak-to-peak range of the corresponding mean spectrum.

**Sampling index i**	**RMSE (a.u.)**	**Range-normalized RMSE (%)**
1	0.1574	0.612
2	0.1291	0.492
3	0.1217	0.468
4	0.1062	0.409
5	0.1148	0.465
6	0.1039	0.434
7	0.1081	0.449
8	0.1078	0.454
9	0.1294	0.553
10	0.1100	0.468
11	0.1608	0.630
12	0.1718	0.681
13	0.1265	0.490
14	0.1806	0.700
15	0.1263	0.492
16	0.1695	0.656
17	0.1482	0.594
18	0.2844	1.152
19	0.3278	1.370
20	0.5980	2.617
21	0.6168	2.675
22	0.1438	0.849
Median	0.1366	0.574
Maximum	0.6168	2.675

3 Reference MATLAB implementations

Listings 1 and 2 provide compact MATLAB reference implementations of the two deterministic spectral subset-selection algorithms used in the benchmark, namely CVQ [36] and KS [40] (the reference numbers refer to the bibliography of the main manuscript). The functions operate on a spectral feature matrix $X \in \mathbb{R}^{p \times n}$, where columns correspond to candidate spectra linked to candidate sampling time points. The returned indices refer to these candidate columns and were mapped to the corresponding t_M values for reporting in Tables S2–S3.

Listing 1: Compact MATLAB reference implementation of CVQ-based RGSS selection by greedy mean-discrepancy minimization.

```
function [Xsel, idx, score] = rgss_select_cvq(X, k, ncomp)
%RGSS_SELECT_CVQ  Greedy CVQ subset selection by mean discrepancy minimization.
%   X      : p x n spectral feature matrix (p variables, n candidate spectra)
%   k      : number of spectra to select
%   ncomp  : number of PCA components used for bandwidth estimation (default: 4)
%   Xsel   : p x k selected spectra
%   idx    : selected candidate indices (1-based)
%   score  : PCA scores of all candidate spectra, returned for visualization

    if nargin < 3 || isempty(ncomp)
        ncomp = 4;
    end

    [p, n] = size(X);
    if k < 1 || k > n
        error('rgss_select_cvq: k must satisfy 1 <= k <= n.');
    end

    ncomp = min([ncomp, p, n-1]);
    [~, score, latent] = pca(X', 'NumComponents', ncomp);
    Sigma = diag(latent(1:ncomp));

    alphaScott = n^(-1/(p + 4));
    h = det((alphaScott^2) * Sigma);

    D2 = squareform(pdist(X', 'squaredeuclidean'));
    Phi = exp(-D2 ./ h);
    phiSum = Phi * ones(n, 1);

    z = false(n, 1);
    for iter = 1:k
        obj = inf(n, 1);
        for j = 1:n
            if z(j)
                continue;
            end
            z(j) = true;
            obj(j) = (double(z)' * Phi * double(z)) / k^2 ...
                    - 2 * (phiSum' * double(z)) / (n * k);
            z(j) = false;
        end
        [~, jBest] = min(obj);
        z(jBest) = true;
    end

    idx = find(z);
    Xsel = X(:, idx);
end
```

Listing 2: Compact MATLAB reference implementation of Kennard–Stone spectral subset selection.

```
function [Xsel, idx, score] = rgss_select_ks(X, k, ncomp)
%RGSS_SELECT_KS  Kennard-Stone subset selection in spectral feature space.
%   X      : p x n spectral feature matrix (p variables, n candidate spectra)
%   k      : number of spectra to select
%   ncomp  : number of PCA components returned for visualization (default: 4)
%   Xsel   : p x k selected spectra
%   idx    : selected candidate indices (1-based, sorted)
%   score  : PCA scores of all candidate spectra, not used for selection

    if nargin < 3 || isempty(ncomp)
        ncomp = 4;
    end

    [p, n] = size(X);
    if k < 1 || k > n
        error('rgss_select_ks: k must satisfy 1 <= k <= n.');
    end

    ncomp = min([ncomp, p, n-1]);
    if ncomp >= 1
        [~, score] = pca(X', 'NumComponents', ncomp);
    else
        score = zeros(n, 1);
    end

    D = squareform(pdist(X', 'euclidean'));

    if k == 1
        [~, idx] = max(mean(D, 2));
        Xsel = X(:, idx);
        return;
    end

    Dupper = triu(D, 1);
    [~, linIdx] = max(Dupper(:));
    [i1, i2] = ind2sub([n, n], linIdx);

    selected = false(n, 1);
    selected([i1, i2]) = true;
    idx = [i1; i2];

    while numel(idx) < k
        pool = find(~selected);
        minDist = min(D(pool, idx), [], 2);
        [~, bestLocal] = max(minDist);
        newIdx = pool(bestLocal);
        selected(newIdx) = true;
        idx(end+1, 1) = newIdx; %#ok<AGROW>
    end

    idx = sort(idx);
    Xsel = X(:, idx);
end
```
